# Measuring aesthetic emotions: A review of the literature and a new assessment tool

**DOI:** 10.1371/journal.pone.0178899

**Published:** 2017-06-05

**Authors:** Ines Schindler, Georg Hosoya, Winfried Menninghaus, Ursula Beermann, Valentin Wagner, Michael Eid, Klaus R. Scherer

**Affiliations:** 1 Department of Education and Psychology, Freie Universität Berlin, Berlin, Germany; 2 Department of Language and Literature, Max Planck Institute for Empirical Aesthetics, Frankfurt am Main, Germany; 3 Department of Psychology, University of Innsbruck, Innsbruck, Austria; 4 Department of Psychology, University of Geneva, Geneva, Switzerland; Leiden University, NETHERLANDS

## Abstract

Aesthetic perception and judgement are not merely cognitive processes, but also involve feelings. Therefore, the empirical study of these experiences requires conceptualization and measurement of aesthetic emotions. Despite the long-standing interest in such emotions, we still lack an assessment tool to capture the broad range of emotions that occur in response to the perceived aesthetic appeal of stimuli. Elicitors of aesthetic emotions are not limited to the arts in the strict sense, but extend to design, built environments, and nature. In this article, we describe the development of a questionnaire that is applicable across many of these domains: the Aesthetic Emotions Scale (Aesthemos). Drawing on theoretical accounts of aesthetic emotions and an extensive review of extant measures of aesthetic emotions within specific domains such as music, literature, film, painting, advertisements, design, and architecture, we propose a framework for studying aesthetic emotions. The Aesthemos, which is based on this framework, contains 21 subscales with two items each, that are designed to assess the emotional signature of responses to stimuli’s perceived aesthetic appeal in a highly differentiated manner. These scales cover prototypical aesthetic emotions (e.g., the feeling of beauty, being moved, fascination, and awe), epistemic emotions (e.g., interest and insight), and emotions indicative of amusement (humor and joy). In addition, the Aesthemos subscales capture both the activating (energy and vitality) and the calming (relaxation) effects of aesthetic experiences, as well as negative emotions that may contribute to aesthetic displeasure (e.g., the feeling of ugliness, boredom, and confusion).

## Introduction

How does beauty feel? The notion that aesthetic appeal is more felt than known has a substantial tradition in philosophical aesthetics. Emotions accompany and inform our experiences of art, literature, music, nature, or appealing sights, sounds, and trains of thought more generally. Consequently, empathetic and affective responses play a central role in accounts of how visual art [1–3], music [4, 5], literature [6, 7], film and television [8–10], art in general [11–15], consumer products [16–19], or natural and built environments [20–22] are processed. However, despite this high relevance of emotions for understanding aesthetic appeal, we still lack a generally agreed-upon classification of and measurement tool for such emotions. This article describes the theoretical and empirical development of a domain-general self-report questionnaire to assess the spectrum of emotions occurring in response to the perceived aesthetic appeal of stimuli. In addition to assessing emotions elicited by literature, music, visual art, and film, the new measure is also applicable to aesthetic experiences beyond the arts in a strict sense, such as emotional responses to nature (e.g., landscapes, plants, or animals), physical attractiveness, design, or consumer products. In the following, we refer to all these kinds of emotions as *aesthetic emotions*.

### Characterizing aesthetic emotions

Aesthetic emotions are the emotions that can arise when a person perceives and evaluates a stimulus for its aesthetic appeal or virtues [23]. Beyond this basic definition, opinions on the precise characterization and range of aesthetic emotions diverge [2, 5, 13, 24–33]. The existing literature does not offer a definition of aesthetic emotions based on a set of individually necessary and jointly sufficient features. It also does not seem likely that such a definition can be found, because the concept of aesthetic emotion—like the more general concept of emotion [34, 35]—is prototypically organized and lacks sharp boundaries. On the one hand, many emotions that can occur in response to a stimulus’s perceived aesthetic appeal can also occur in response to stimuli that are appreciated for non-aesthetic reasons. On the other hand, it may be debated whether some states elicited by perceived aesthetic virtues are emotions at all. Membership in the category *aesthetic emotions* is thus a matter of degree: the degree of similarity to the prototypical aesthetic emotions.

While the boundaries of the category *aesthetic emotions* remain fuzzy, scholars largely agree on the prototype for aesthetic emotions. We have identified some of its features that we consider central. First, we label as aesthetic emotions only emotions that recipients actually feel, rather than emotions that are represented, expressed, or alluded to in the respective stimuli (see, e.g., [36, 37], on the difference between emotion perception and emotion induction).

Second, in contrast to utilitarian emotions [32, 33], the intrinsic aesthetic appeal of a stimulus rather than its instrumentality for achieving personal goals elicits aesthetic emotions. This consideration is particularly important when studying aesthetic emotions outside the traditional arts, such as responses to consumer products. For instance, Desmet [16] identified six sources of emotions in human-product interactions: (1) the material qualities of the product, (2) personal meanings associated with the product, (3) interactive qualities of using the product, (4) activities enabled or facilitated by the product, (5) ourselves as users or owners of the product, and (6) ourselves when others apply the product to us. It is clear from this list that some emotional reactions to products are linked to the past, present, or future usefulness of the product for achieving goals and thus can be regarded as utilitarian emotions. In contrast, aesthetic emotions do not reflect an interest in using a product but rather an interest in the product per se. Along the same lines, Chatterjee and Vartanian [11] have suggested that aesthetic emotions are triggered by objects rather than outcomes, a contrast that may also be reflected in the activity of two dissociable neural systems. Aesthetic, object-related emotions correspond to activity in the liking system, while outcome-related (utilitarian) emotions correspond to activity in the wanting system [38, 39].

Accordingly, the subjective experience and savoring of aesthetic emotions takes precedence over the emotions’ signaling value for preparing goal-directed actions (other than prolonged or repeated exposure to the stimulus). Nevertheless, it is difficult to draw a clear distinction between aesthetic and other emotions in our experiences. For example, perceived beauty also enhances the perceived social and intellectual competence of people [40, 41], the perceived usability of products [42, 43], and the perceived correctness of solutions to simple mathematical addition problems [44]: what is beautiful seems good, usable, and true.

Third, the field of aesthetics is traditionally limited to perceptual input from the distance senses [45]. Consequently, aesthetic emotions are elicited through vision, hearing, and cognitive processing in response to such input. The contact senses of touch, taste, and smell clearly give rise to emotions [46, 47], but these are not in a strict sense aesthetic emotions. This also ties in with the above considerations about viewing versus using a product. It is often impossible to use a product without receiving tactile information—and frequently gustatory and olfactory information as well.

Fourth, aesthetic emotions are intertwined with aesthetic judgment [2, 23]. On the one hand, several studies have shown that the cognitive evaluation of a stimulus as art or non-art informs the emotions that are felt and reported in response to it [48–51]—although, as illustrated by responses to beauty in nature, such top-down art framing is by no means a necessary ingredient of aesthetic emotions.

On the other hand, aesthetic emotions play an epistemic role in aesthetic judgment (see, e.g., [52]): a person’s felt appreciation of a stimulus serves as an indicator of its perceived aesthetic appeal. To be sure, aesthetic judgment can be influenced by any conceivable emotion. In fact, people’s aesthetic pleasure and resulting aesthetic judgment can be driven by and confused with aesthetically irrelevant factors (such as pleasure associated with status, conformity, or familiarity; cf. [52]). However, some emotions cannot reasonably be attributed to the form or content of the stimulus that is being aesthetically evaluated. For instance, emotions like envy and pride are not elicited by objects per se but rather by a person’s values and motivations when interacting with the surrounding social context. The label *aesthetic emotion* is typically limited to emotions that result from a stimulus’s form or content and thus provide input that is deemed relevant and appropriate for aesthetic judgment.

In sum, aesthetic emotions are aesthetically evaluative emotions [23] because they influence and are influenced by aesthetic judgment. The aesthetic evaluation of stimuli both informs and results from the experience and regulation of aesthetic emotions.

This prototype-based framework for defining aesthetic emotions helped us conduct the present research. However, because characterizing a prototype does not serve to delimit the entire domain to be studied, we sought to derive an operational definition of aesthetic emotions. To that end, we used a combined top-down theoretical and bottom-up empirical approach to identify emotions that need to be considered. We identified emotions that are labelled *aesthetic* in theoretical treatments and emotions that have been included in the assessment of aesthetic emotions and experience. Moreover, we consulted empirical studies on the words that are used to designate aesthetic appeal dimensions of stimuli such as visual art, music, literature, environments, and consumer products.

Our aim was to develop a domain-general measure of aesthetic emotions that is comprehensive yet parsimonious. Therefore, we were careful to also consider emotions that do not bear a strong similarity to the prototypical aesthetic emotions (or even the prototypical emotions) but have nevertheless been included among the aesthetic emotions in some publication. We developed the initial candidate items for our new instrument based on an extensive review of the literature. In order to determine which items should be included in the final version of the questionnaire, we then conducted a field study involving people who had just attended a film screening, a theater performance, a concert, an art exhibition, or some other event of aesthetic interest.

### Measures of aesthetic emotions and aesthetic experience

Various published studies include assessments of aesthetic emotions but are limited in one of two ways. First, researchers have employed general measures of emotion. These were developed to assess basic emotions or emotion dimensions like valence and arousal rather than specific emotions that are relevant to experiences of the aesthetic appeal of stimuli (for overviews, see [53] for marketing, [19, 54, 55] for consumer products, [56, 57] for advertising, and [58] for music). Two of the most widely employed discrete emotion models are those of Izard [59] and Plutchik [60]. The Positive and Negative Affect Schedule (PANAS [61]) is a frequently used dimensional measure.

However, such general emotion measures may not capture the full spectrum of emotions experienced in response to perceived aesthetic virtues of stimuli [16, 19, 58, 62]. Crucially, general models include far more negative emotions than positive ones. As a result, they may not offer a sufficiently nuanced basis to account for the great variety of positive aesthetic emotions.

Moreover, in the case of negatively valenced emotions, appreciable aesthetic experiences are by no means limited to pure and strong feelings of, for instance, sadness [63–65]. Rather, otherwise appealing stimuli that evoke such negative emotions also include positive affective antidotes (see [66] for a detailed analysis of a sad film clip), which results in complex or mixed emotional states like being moved or suspense [64]. Indeed, recent studies have shown that aesthetic liking of sad film scenes [63, 67] and sad music [68, 69] is linked to feelings of being moved/touched, nostalgia, or tenderness rather than mere sadness [63, 67]. To differentiate such subtle, mixed emotional states from purely negative emotions, it is necessary to move beyond general models of basic emotions or global dimensions of affect. That is, we need to draw upon the rich emotion vocabulary that is available to characterize enjoyable variants of basically negative or mixed emotions (e.g., feeling touched, nostalgic, fascinated, or awestruck).

The second limitation of extant measures of aesthetic emotions is their domain-specificity. As the overview in Table 1 reveals, measures of aesthetic emotions and experiences capture the richness and subtlety of these emotions but are typically specific to a single aesthetic domain such as music, dance, painting, film/television, literature, consumer products, or environments. Therefore, it is likely that the resulting scales cannot readily be employed in other contexts [19, 54, 56]. For instance, while humor and fear have not figured prominently among self-reported musical emotions [58, 70] (but see [71–73] for studies that include humor), it would be difficult to assess the emotional impact of comedies or thrillers without reference to these emotions.

**Table 1:** Overview of measures of aesthetic emotions and experience.

Measure	Domain	Description	Authors/References
Hevner adjective checklist	Music	8 (Hevner; numbers) or 9 (Schubert; letters) clusters with 2–11 items for describing emotions expressed in music: (1/G) (e.g., spiritual, sacred), (2/F) (e.g., sad, melancholy), (3/D) (e.g., dreamy, sentimental), (4/C) (e.g., serene, soothing), (5/B) (e.g., humorous, playful), (6/A) (e.g., happy, cheerful), (7/H) (e.g., exciting, dramatic), (7/I) (e.g., agitated, restless), (8/G) (e.g., vigorous, majestic), (E) (tragic, yearning)	Hevner [72]; Schubert [74]
9-Affective Dimensions (9-AD)	Music	41 items, 9 dimensions (3–5 items each): (1) evil (e.g., anger, frustrated), (2) sensual (e.g., tender, beautiful), (3) potency (e.g., heroic, majestic), (4) humor (e.g., amused, playful), (5) pastoral (e.g., calm, relaxed), (6) longing (e.g., yearning, longing), (7) depression (e.g., depressed, sad), (8) sedative (e.g., reflective, serene), (9) activity (e.g., determined, vigorous)	Asmus [71]
Geneva Emotional Music Scale (GEMS)	Music	3 versions (ultra-short, short, and full with 9–45 items), all measuring 9 emotion factors: (1) wonder (e.g., filled with wonder, allured), (2) transcendence (e.g., feeling of transcendence, thrills), (3) tenderness (e.g., affectionate, mellowed), (4) nostalgia (e.g., sentimental, dreamy), (5) peacefulness (e.g., calm, serene), (6) power (e.g., triumphant, strong), (7) joyful activation (e.g., joyful, animated), (8) tension (e.g., agitated, tense), (9) sadness (e.g., sad, tearful)	Zentner, Grandjean, & Scherer [70]; Zentner & Eerola [58]
Geneva Music-Induced Affect Checklist (GEMIAC)	Music	Brief measure for rapid assessment of affect states to complement the GEMS; 14 emotion categories assessed with 14 fuzzy category sets, each defined by two affective terms; includes the 9 emotion categories of the GEMS and 5 additional categories: (1) being moved (moved, touched), (2) enthusiasm (inspired, enthusiastic), (3) energy (energetic, lively), (4) disengagement (indifferent, bored), (5) anger (agitated, aggressive)	Coutinho & Scherer [75]
Questionnaire to measure prevalence of musical emotions	Music	Measure to assess emotional responses to music in a closed and open-ended format 44 emotion terms to assess frequency of emotional responses, with the 10 most prevalent emotions being: happy, enjoying, relaxed, calm, amused, moved, nostalgic, loving, interested, and longing Open responses characterizing the emotion felt during a recent episode of music listening yielded the 10 most frequent categories: happy-elated, sad-melancholic, calm-content, nostalgic-longing, aroused-alert, angry-irritated, loving-tender, moved-touched, interested-expectant, and proud-confident	Juslin, Liljeström, Laukka, Västfjäll, & Lundqvist [73]
Instrument for measuring aesthetic experience of dance performances	Dance	Measure to assess dancers’ and spectators’ aesthetic experience; 35 items, 3 factors: (1) dynamism (e.g., expressive, powerful, strong, exciting), (2) exceptionality (e.g., eternal, ineffable, unique, exceptional), (3) affective evaluation (e.g., subtle, elegant, seductive, sensitive)	Vukadinović & Marković [76]
Scales to measure readers’ perceptions of emotions in a story and their emotional experience	Literature	3 blocks of scales (5–15 items per block): (1) fiction-based emotions with 2 factors: (F1) sympathy (sympathy, understanding), (F2) involvement (study 1: e.g., pity, awe; study 2: disgust, mockery), (2) artefact emotions with 3 factors: (F1) attractiveness (e.g., good, captivating, beautiful, amusing), (F2) novelty (e.g., original, strange), (F3) comprehensibility (e.g., comprehensible, readable), (3) perceived emotions of story characters: 15 items (e.g., rebellious, sad, terrified, confused)	Andringa [77]
Fragebogen zum Leseerleben (FBLE) [Reading Experience Questionnaire]	Literature	77 items, 14 factors (4–10 items each): (1) focusing of attention (e.g., thoughts wandering during reading, thought of something else while reading), (2) immersion in a text (e.g., forgot the world around me, time was flying), (3) vividness (e.g., could picture the characters, text seemed rather abstract [reversed]), (4) being there (e.g., felt transported in the world described in text, reading was like a journey to a different place), (5) ending of reception (e.g., text stayed with me after reading, was easy to concentrate on other things after reading [reversed]), (6) suspense (e.g., was curious to learn what happens next, text was boring [reversed]), (7) emotional involvement (e.g., text touched me emotionally, text evoked real emotions), (8) pleasure in reading (e.g., reading the text was fun, I liked the text), (9) identification (e.g., I found the protagonists’ actions admirable, I felt sorry for the protagonist), (10) parasocial interaction (e.g., I would have liked to talk to the protagonist, I would like to read another text with this protagonist), (11) cognitive involvement (e.g., text occupied thoughts, text relevant to my everyday life), (12) thematic interest (e.g., topic of text is personally important, text concerns questions which I have often thought about), (13) analytic mode of reception (e.g., during reading I paid attention to whether everything in the text fits together, I paid attention to the style of the text), (14) ease of cognitive access (e.g., it was easy to follow the story line, I was unsure whether I understood everything [reversed])	Appel, Koch, Schreier, & Groeben [78]; see also Rössler [79] (pp. 74–82)
Scales to measure affective responses during reading	Literature	26 items, 6 scales (3–6 items each): Narrative feelings (1) sympathy/empathy (e.g., I felt understanding for the narrator, I felt pity for the narrator), (2) identification (e.g., I could recognize myself in the narrator, it was like I was looking through the eyes of the narrator), (3) absorption (e.g., I felt absorbed in the story, I felt involved in the events), (4) empathic distress (e.g., story made me feel miserable, story made me feel sad); Aesthetic Feelings: (5) attractiveness (e.g., interesting, beautiful, captivating), (6) foregrounding (surprising, striking, original)	Koopman [80]
Questionnaire representing viewpoints from sociology, psychology, and theater studies	Theater	3 blocks of assessment (in addition to sociological variables): (1) Complexity and conventionality, containing 32 items measuring 4 scales (8 items each): (S1) complexity (e.g., the scene contained much information, gave new insights), (S2) noncomplexity (e.g., the scene had a clear meaning, I could follow the story easily), (S3) unconventionality (e.g., the portrayed situation was bizarre, the behavior of the characters was strange), (S4) conventionality (e.g., this scene displayed common norms and values, the characters behaved realistically); (2) Identification, containing 16 emotions measuring 2 scales (8 items each): (S1) negative emotions (e.g., disgust, fear, anger, sadness), (S2) positive emotions (e.g., in love, pleasure, desire, tension); (3) Emotions, containing 16 items measuring 3 scales (4–7 items each): (S1) empathic emotions (e.g., pity, involvement, affection, being touched), (S2) positive task emotions (e.g., excitement, admiration, inspiration, challenge), (S3) negative task emotions (confusion, irritation, boredom, listlessness)	Konijn [81]
Modified version of the Differential Affect Scale (M-DAS)	Film/ Television	48 items, 16 scales (3 items each): (1) pleasure, (2) joy, (3) contentment, (4) love, (5) fascination, (6) enchantment, (7) interest, (8) surprise, (9) sadness, (10) anger, (11) disgust, (12) contempt, (13) fear, (14) boredom, (15) shame, (16) guilt	Renaud & Unz [82]
Narrative Engagement Scale	Film/ Television	12 items, 4 scales (3 items each): (1) narrative understanding (all reversed: hard time making sense of what was going on, understanding of characters is unclear, hard time recognizing thread of story), (2) attentional focus (all reversed: mind wandering during program, found myself thinking of other things, hard time keeping my mind on program), (3) narrative presence (mind was inside the world created by story, program created a new world, story world closer to me than real world), (4) emotional engagement (story affected me emotionally, felt happy and sad with main characters, felt sorry for some of the characters)	Busselle & Bilandzic [83]
Emotional Gratification Scales	Film/ Television	28 items, 7 factors (4 items each): (1) fun (e.g., makes me laugh, puts me in a good mood, amuses me), (2) thrill (e.g., like the adrenalin kick, enjoy the excitement, like the tension), (3) empathic sadness (e.g., like being moved to tears, enjoy when I can cry, like sad and poignant moments), (4) contemplative experiences (e.g., makes me think about important topics, gives me new insights, makes me think about myself), (5) character engagement (e.g., like empathizing with the characters, like taking the role of the characters, like living through things together with the characters)	Bartsch [84]
List of emotion adjectives	Painting	23 scales comprising 1–5 emotion adjectives to describe emotional reactions to painting reproductions: (1) calmness (e.g., peace, serenity), (2) depression (e.g., sadness, melancholy), (3) solitude, (4) fatigue, (5) excitement (e.g., anxiety, restlessness), (6) frenzy, (7) tumult (e.g., agitation, confusion), (8) surprise, (9) wanderlust (e.g., adventurous, yearning), (10) wonder (e.g., curiosity), (11) delight (e.g., happiness, pleasant), (12) exaltation (e.g., exhilaration, ecstasy), (13) sprightly (e.g., vivacious, cheerful), (14) romantic, (15) awe (e.g., sublimity, majestic), (16) apathy (e.g., laziness, stupor), (17) sympathy, (18) unpleasantness, (19) love, (20) fear, (21) anger, (22) hate, (23) admiration	Israeli [85]
Survey for the Assessment of Aesthetic Perception (SAAP)	Visual art (painting/ sculpture)	16 items, 3 scales (4–7 items each): (1) cognition (e.g., have to think about artwork, exciting, content of artwork occupies my mind, provides me with new information), (2) emotion (relaxed, feel good, feel fresh, feel colorful), (3) self-congruency (e.g., reconsidering my personal life, remember my life history, discover new aspects of myself, artwork has something to do with myself)	Rowold [86]
Measure of the affective and cognitive components involved in the perception of visual art	Painting	35 items, 9 factors (3–5 items each): Emotion factors: (1) negative emotion, high arousal (unease, anxiety, uncertainty, disquiet), (2) negative emotion, low arousal (sadness, despair, gloom, loneliness), (3) positive emotion, high arousal (excitement, enthusiasm, thrill), (4) positive emotion, low arousal (happiness, joy, gladness, serenity); Cognitive factors: (5) curiosity (interesting, arousing curiosity, fascinating, intellectually stimulating), (6) aesthetic (aesthetically, attractive, beautiful, appealing), (7) creativity (original, distinct, creative, inventive), (8) skill (workmanship, well crafted, skillfully made); Evaluation index: (9) evaluation (good, positive, favorable, pleasing, like)	Hagtvedt, Hagtvedt, & Patrick [87]
List of descriptors of the aesthetic experience and emotional content of paintings	Painting	31 items, 2 factors: (1) affective tone (e.g., lovely, charming, cheerful, scary [reversed]), (2) aesthetic experience (e.g., exceptional, profound, unique, awing)	Marković [30]
Art Reception Survey (ARS)	Painting	29 items, 6 factors (4–5 items each): (1) cognitive stimulation (e.g., makes me curious, is thought-provoking, exciting to think about, is fun to deal with), (2) negative emotionality (e.g., makes me feel afraid, makes me sad, makes me feel troubled, makes me feel lonesome), (3) expertise (e.g., can relate painting to its art historical context, can relate painting to a particular artist, know this painting, have an idea what artist is trying to convey), (4) self-reference (makes me think about my own life history, can associate painting with personal biography, personal memories linked to painting, painting mirrors own personal emotional state), (5) artistic quality (e.g., is unique, features a high level of creativity, composition is of high quality, artist’s manner of painting is fascinating), (6) positive attraction (e.g., is pleasant, is beautiful, thrills me, feel inspired by this painting)	Hager, Hagemann, Danner, & Schankin [88]
Wordlist for the assessment of emotions in response to modern art	Modern art pictures	19 items, 3 factors (6–7 items each): (1) liking/interest (e.g., pleased, sympathetic, fascinated, ruminative), (2) negative affect (e.g., uneasy, threatened, pessimistic, stressed), (3) repulsion/aggression (e.g., angry, aggressive, repulsive, disgusting)	Ortner [89]; Panagl [90]
Questionnaire on subjective aesthetic experiences	Fine art museum exhibition	The self-report measure covers emotions evoked by an artwork, aesthetic evaluations, and general appraisal of an artwork; it is part of an integrative methodology also assessing locomotion and physiological data; 19 items, 5 factors: (1) aesthetic quality (e.g., pleasing, beautiful, emotionally moving), (2) surprise/humor (e.g., surprising, makes one laugh, makes one think), (3) negative emotion (e.g., work conveys sadness, fear, anger), (4) dominance (e.g., work experienced as dominant, stimulating), (5) curative quality (e.g., work is well staged and hung, suitable in the context of other artworks)	Tröndle & Tschacher [91]; Tschacher et al. [92]
Aesthetic Experience Scale (AES)	Multiple art domains	28 items, 5 factors (4–7 items each): (1) cognitive synergies and elaboration (e.g., to appreciate a poem more when the form enhances its meaning, to realize that the knowledge of weaving increases your enjoyment of tapestry), (2) emotional closeness (e.g., to feel fulfilled when surrounded by beautiful things made by you, to like a picture because of your color preferences), (3) experiential emotional distancing (e.g., to go away with a smile of pleasure when looking at an everyday scene, to forget time when participating in aesthetic activities), (4) paratelic mode (e.g., to feel excited when trying to compose music or paint something, to feel emotionally enhanced and fulfilled after appreciating an artwork), (5) expressive perception (e.g., to enjoy trying to identify feelings on faces in portraits, to feel completely absorbed in a work of art or music)	Stamatopoulou [93]
Aesthetic Experiences Scale/Unusual Aesthetic Emotions Scale	Multiple art domains	10 items, 3 factors (2–5 items each): (1) chills (feel chills down your spine, feel like your hair is standing on end, get goose bumps), (2) feeling touched (feel touched, feel like crying), (3) absorption (e.g., feel absorbed and immersed, completely lose track of time, feel like you’re somewhere else)	Silvia & Nusbaum [94]; Silvia, Fayn, Nusbaum, & Beaty [95]
Feelings inventory	Advertise-ments	69 items, 3 factors (13–32 items each): (1) upbeat (e.g., active, cheerful, humorous, inspired, proud, satisfied), (2) negative (e.g., angry, bored, disgusted, lonely, sad, suspicious), (3) warm (e.g., affectionate, calm, contemplative, moved, sentimental)	Edell & Burke [96]
Measurement of emotional responses to advertisements	Advertise-ments	Identified 24 factors (F; Holbrook & Batra) and 20 clusters (C; Batra & Holbrook) Matching factors/clusters: (F1/C12) joy/elation (e.g., happy, delighted, pleased), (F3/C16) sadness (e.g., sad, sorrowful), (F6/C17) contempt/scornful (e.g., scornful, contemptuous, disdainful), (F7/C10) fear (e.g., fearful, afraid), (F10/C14) affection/social affection (e.g., loving, affectionate), (F11/C1) activation (aroused, active, excited), (F13/C9) hypoactivation/drowsy (e.g., drowsy, sluggish), (F14/C4) competence/confidence (confident, in control, competent), (F15/C6) helplessness/dominated (e.g., powerless, dominated), (F16/C20) surgency (playful, entertained, lighthearted), (F17/C3) skepticism (e.g., skeptical, suspicious), (F19/C7) serenity/restful (e.g., restful, serene), (F21/C11) desire (e.g., desirous, wishful, full of craving), (F22/C13) duty/moral (e.g., moral, virtuous), (F24/C15) gratitude (e.g., grateful, thankful) Mismatching factors/clusters: (F2) surprise (surprised, amazed, astonished), (F4) anger (e.g., angry, irritated, enraged), (F5) disgust (e.g., disgusted, revolted, full of loathing), (F8) shame (ashamed, embarrassed, humiliated), (F9) guilt (guilty, remorseful, regretful), (F12) hyperactivation (panicked, confused, overstimulated), (F18) pride (proud, superior, worthy), (F20) conflict (tense, frustrated, conflictful), (F23) faith (reverent, worshipful, spiritual); (C2) tension (tense, distressed, anxious), (C5) anger (angry, enraged, mad), (C8) bored (e.g., bored, unimpressed, unexcited), (C18) irritation (disgusted, irritated, annoyed), (C19) soothed (soothed, spiritual)	Holbrook & Batra [97]; Batra & Holbrook [98]
Feeling responses to advertising	Advertise-ments	31 feeling clusters: 16 positive feeling clusters: (1) playful/childish, (2) friendly, (3) humorous, (4) delighted, (5) interested, (6) strong/confident, (7) warm/tender, (8) relaxed, (9) energetic/impulsive, (10) eager/excited, (11) contemplative, (12) pride, (13) persuaded/expectant, (14) vigorous/challenged, (15) amazed, (16) set/informed 15 negative feeling clusters: (1) fear, (2) bad/sick, (3) confused, (4) indifferent, (5) bored, (6) sad, (7) anxious, (8) helpless/timid, (9) ugly/stupid, (10) pity/deceived, (11) mad, (12) disagreeable, (13) disgusted, (14) irritated, (15) moody/frustrated	Aaker, Stayman, & Vezina [99]
Affective reactions to apparel advertisements	Advertise-ments	14 items, 5 factors (2–3 items each): (1) negative feeling (humiliated, distasteful, offended), (2) sensual feeling (erotic, sexy, sensual), (3) upbeat feeling (merry, energetic, vigorous), (4) warm feeling (warmhearted, sentimental, warm), (5) dull feeling (bored, dull)	Oh [56]
Consumption Emotion Set (CES)	Consumer products	47 items, 16 scales (2–3 items each) and 4 single items: (1) anger (frustrated, angry, irritated), (2) discontent (unfulfilled, discontented), (3) worry (nervous, worried, tense), (4) sadness (depressed, sad, miserable), (5) fear (scared, afraid, panicky), (6) shame (embarrassed, ash amed, humiliated), (7) envy (envious, jealous), (8) loneliness (lonely, homesick), (9) romantic love (sexy, romantic, passionate), (10) love (loving, sentimental, warm hearted), (11) peacefulness (calm, peaceful), (12) contentment (contented, fulfilled), (13) optimism (optimistic, encouraged, hopeful), (14) joy (happy, pleased, joyful), (15) excitement (excited, thrilled, enthusiastic), (16) surprise (surprised, amazed, astonished); additional single items: guilty, proud, eager, relieved	Richins [19]
Product Emotion Measurement Instrument (PrEmo/PrEmo2)	Consumer products	Non-verbal self-report instrument measuring emotions with the use of expressive cartoon animations 14 emotions of PrEmo: 7 pleasant emotions: (1) desire, (2) pleasant surprise*, (3) inspiration*, (4) amusement, (5) admiration, (6) satisfaction, (7) fascination; 7 unpleasant emotions: (8) indignation*, (9) contempt, (10) disgust, (11) unpleasant surprise*, (12) dissatisfaction, (13) disappointment*, (14) boredom; *not included in PrEmo2 new emotions in PrEmo2: pride, hope, shame, fear, sadness	PrEmo: Desmet [100]; PrEmo2: Laurans & Desmet [101]
Pre-Purchase Emotion Set	Consumer products	18 pre-purchase affects (single items): (1) amazed, (2) cheerful, (3) concerned, (4) contented, (5) delighted, (6) encouraged, (7) enthusiastic, (8) excited, (9) fulfilled, (10) glad, (11) good, (12) happy, (13) hopeful, (14) interested, (15) joyful, (16) pleased, (17) surprised, (18) thrilled	Seva, Helander, & Duh [102]
Questionnaire on positive emotions during human-product interactions	Consumer products	25 positive emotions experienced in response to (using) consumer products (ordered by decreasing frequency of occurrence): (1) joy, (2) satisfaction, (3) amusement, (4) relaxation, (5) love, (6) confidence, (7) desire, (8) energized, (9) fascination, (10) kindness, (11) inspiration, (12) pleasant surprise, (13) anticipation, (14) respect, (15) sympathy, (16) pride, (17) admiration, (18) hope, (19) enchantment, (20) courage, (21) euphoria, (22) relief, (23) dreaminess, (24) lust, (25) worship	Desmet [16]
Consumption emotion measurement scale	Full-service restaurants	32 items, 4 factors (3–14 items each): (1) excitement (e.g., excited, surprised, amazed, curious), (2) comfort (e.g., comfortable, contented, friendly, relaxed), (3) annoyance (e.g., irritated, frustrated, disappointed, anger), (4) romance (romantic, love, sentimental)	Han, Back, & Barrett [54]; Modification for upscale restaurants: Han & Jeong [103]
Scales of the Affective Quality Attributed to Places	Built and natural environments	40 items, 8 scales (5 items each), forming 4 bipolar scales: (1) arousing quality (e.g., intense, arousing, active) and reversed sleepy quality (e.g., inactive, drowsy, idle), (2) exciting quality (e.g., exhilarating, stimulating, interesting) and reversed gloomy quality (e.g., dreary, dull, boring), (3) pleasant quality (e.g., pleasing, pretty, beautiful) and reversed unpleasant quality (e.g., dissatisfying, repulsive, uncomfortable), (4) distressing quality (e.g., frenzied, tense, panicky) and reversed relaxing quality (e.g., serene, peaceful, calm)	Russell & Pratt [104]
Affect scales measuring experience of places	Urban landscapes	6 items: (1) comfortable, (2) excited/stimulated, (3) distressed/anxious, (4) bored, (5) relaxed, (6) safe	Galindo & Rodríguez [105]
Destination Emotion Scale (DES)	Tourist destinations	15 items, 3 factors (5 items each): (1) joy (cheerful, pleasure, joy, enthusiasm, delight), (2) love (tenderness, love, caring, affection, warm-hearted), (3) positive surprise (amazement, astonishment, fascinated, inspired, surprise)	Hosany & Gilbert [106]

To identify the spectrum of aesthetic emotions, we searched the literature for questionnaires assessing such emotions or responses to perceived aesthetic virtues of stimuli more generally. Table 1 presents measures that we considered when developing and selecting items for our scale. In addition, we wanted to provide an encompassing overview of the state of the research measuring aesthetic emotions. Therefore, we have also included measures that we identified in an additional in-depth literature search after we conducted our study (some of these measures were published after our data collection).

When compiling Table 1, we limited our search to measures that were developed or considerably modified specifically for studies of aesthetic perceptions and evaluations. As noted above, general emotion measures do not capture the full spectrum of aesthetic emotions. In addition, we only included measures that assess a range of emotions and are not limited to two or three emotion dimensions like valence and arousal. Most notably, this criterion led to the exclusion of the Pleasure-Arousal-Dominance (PAD) scales [107]. While the PAD scales have been employed in various studies, including studies on the emotional impact of environments [108] and physical attractiveness [109], they do not allow for a differentiated assessment of specific aesthetic emotions or emotion categories. However, we included a conceptually similar measure of four affective dimensions by Russell and Pratt [104] in Table 1, as this measure contains eight scales that can be analyzed individually.

It should be noted that the measures listed in Table 1 are not limited to measures of subjectively felt emotions. Rather, we also included measures of emotions that are represented or expressed in the respective stimuli or of potential emotional effects that are attributed to the stimuli (e.g., when respondents rate whether a film is moving or music is joyful, without also reporting on whether they actually feel these emotions when watching the film or listening to the music). In several cases, the authors—and probably the participants as well—did not clearly distinguish between felt, expressed, and attributed emotions. Even where the distinction was explicitly made, consideration of expressed or attributed emotions is still informative, as these emotions may also be felt by recipients (see [36] on the possible types of relationships that may exist between perceived and induced emotions). That is, although emotions represented or expressed in a stimulus are not aesthetic emotions according to our description, they are still informative with regard to the aesthetic emotions that may be elicited by the stimulus in question.

Compilation of Table 1, and more specifically the measures included there, served two functions. First, it provided us with a rich base of emotion terms from which to select candidate items. Second, sorting through the items and the respective scales, factors, or clusters enabled us to identify broad subclasses of aesthetic emotions that are relevant to all or at least many of the studied domains. We found that the number of subscales, factors, or clusters considered necessary to capture reactions to the perceived aesthetic appeal of stimuli varies considerably, ranging from the two factors identified by Marković [30] to the 31 clusters of Aaker, Stayman, and Vezina [99]. For our purposes, the more fine-grained measures were of greater interest. Aggregating across many different emotion terms typically results in overarching dimensions that cannot capture the specific emotional signatures of the stimuli under study. Focusing on such measures, we drew several conclusions about which specific emotions should or should not be included in a domain-general measure of aesthetic emotions. More specifically, we identified broad subclasses of emotions from which we would or would not select our candidate items; we present these in the following sections.

#### Prototypical aesthetic emotions

Some emotions that typically are not considered in lists of general or basic emotions are highly salient in Table 1. Several measures include emotions like being moved or being touched, fascination, captivation, awe, feelings of transcendence, wonder, and admiration. In the emotion literature, emotions like these (and also inspiration) have been characterized as appreciation [8, 110, 111], other-praising [112, 113], and self-transcendent emotions [114–116]. Most notably for the present context, this group of emotions has also been considered to constitute the prototypical aesthetic emotions [5, 24, 28, 29, 32, 114]. Specifically, scholars who view aesthetic emotions as limited to explicitly aesthetically evaluative emotions or emotions responding to an artwork’s style or execution rather than its content have considered this group to be the genuinely aesthetic emotions (see also [14] on artifact emotions). For instance, Frijda [24] highlighted the fascinating and captivating potential of aesthetic emotions. Marković [29, 30] considered fascination to be central to aesthetic experience and also listed rapture, awe, and admiration as prototypical aesthetic emotions. Similarly, Miall and Kuiken [7] described feeling struck or captured as well as surprise, admiration, and appreciation as aesthetic feelings (as part of a larger category of evaluative feelings) in response to the formal characteristics of a text. Scherer’s [32] list of aesthetic emotions (see also [5]) includes being moved, awed, or full of wonder, rapture, and admiration. Finally, Brady and Haapala [117] have discussed both melancholy and nostalgia as aesthetic emotions.

Notably, the emotions that are considered as prototypical aesthetic emotions are not limited to pleasing elicitors, but rather reflect appreciation irrespective of pleasingness. We want to point out that we deliberately speak of the pleasingness of a stimulus and the resulting emotion as feeling pleased rather than pleasure. We use the term *pleasure* to refer to all kinds of pleasure, including aesthetic pleasure. Aesthetic pleasure (like pleasures of the mind [118] or pleasures derived from sense-making [119, 120] more generally) is not limited to pleasing sensations and purely positive emotions. Rather, aesthetic pleasure results from a well-orchestrated sequence or mix of emotions and sensations, regardless of whether these are of positive, mixed, or negative valence [45, 64, 100]. This point is underscored by the prototypical aesthetic emotions. While all of these emotions indicate aesthetic pleasure, elicitors of being moved [63, 121–123], awe [95, 114, 124], and fascination [30, 125, 126] can have rather unpleasant aspects. Similarly, nostalgia is a mixed, bittersweet emotion, defined as a sentimental longing for the past. While nostalgia can have adaptive functions, which have been characterized as falling into the two broad categories of self-positivity or preserving a sense of self and social connectedness [127, 128], this emotion is double-edged. Nostalgia and also longing can have maladaptive outcomes in terms of lower well-being and coping abilities [129–131].

While Table 1 reveals that appreciative emotions do not suffice to capture the richness of emotions experienced in response to perceived aesthetic virtues (see also [132]), it is clear that these emotions should receive special attention in a measure of aesthetic emotions. To this end, we need to sample the full range of emotions linked to appreciation as well as transcending one’s ordinary level of experience and finding meaning. Table 1 includes a broad variety of such emotions, suggesting that nostalgia, longing, sentimentality, and enchantment need to be considered in addition to awe, wonder, transcendence, being moved, fascination, and admiration.

Moreover, the compilation in Table 1 also points to the necessity to include the feeling of beauty in the realm of aesthetically appreciative emotions. Kant [133] already spoke of a “feeling of beauty” rather than considering the use of aesthetic appreciation terms as providing cognitive evaluations of stimuli. That is, beauty is associated with specific feeling qualities such as feeling oneself in harmony with the aesthetic object. Kant was also the first to label the feeling of beauty or the feeling of the sublime as *aesthetic emotions*.

More recently, other authors have also suggested that people feel rather than know beauty (cf. [134]). In particular, following Kant, Armstrong and Detweiler-Bedell [135] argued that the feeling of beauty reflects the exhilarating “prospect of understanding something novel and particularly meaningful” (p. 305). Thus, they consider the feeling of beauty to be linked to a search for meaning (but to differ from interest and awe), rather than a merely pleasing emotion.

In developing a measure of aesthetic emotions, we therefore considered it important to include feelings of beauty and related aesthetically evaluative terms. As Table 1 does not include a broad variety of such terms, we also consulted studies of terms that are used to designate aesthetic appeal dimensions of various stimuli (as detailed further below).

#### Pleasing emotions and epistemic emotions

Aesthetic pleasure can be accompanied by a variety of experiences and emotions, including pleasing sensory stimulation and elaborate ways of finding meaning in art [2, 3, 110]. James [136] already recognized this distinction. He considered aesthetic emotions to be the immediate and primary sensory pleasure resulting from exposure to a stimulus. Nevertheless, he recognized that secondary pleasures play an additional role in the enjoyment of art.

Beginning with the work of Berlyne [137, 138] and Wohlwill [22], the distinction between pleasingness and meaning (or, respectively, interest/attention) as sources of enjoyment runs through various accounts of aesthetic experience [2, 110, 139, 140]. Cupchik [12] identified two modes of processing: the reactive mode, which is linked to immediate pleasingness or pleasing excitement, and the reflective mode, which is linked to efforts toward meaning and the enjoyment of challenge. Similarly, the processing fluency of aesthetic stimuli and their perceptual challenge both contribute to enjoyment [141–146]. Therefore, it is important to capture the emotions linked to pleasing and challenging modes of aesthetic enjoyment in a measure of aesthetic emotions.

The pleasing emotions are limited to those with positive valence. They can be derived, for instance, from models of hedonic or affect-regulatory motivations for seeking exposure to art and other stimuli of aesthetic interest (for an overview see [147]). Zillmann [148] identified four characteristics of media content that can contribute to hedonic mood regulation: positive hedonic valence, excitatory potential, absorption potential, and lack of semantic affinity with current negative moods. Happiness is the emotion that most clearly fits this description. Other pleasing emotions include (positive) excitement as well as relaxation, playfulness, cheerfulness, and humor [110]. This spectrum of emotions is also well represented in Table 1, with various measures including happiness, cheerfulness, pleasingness, humor, amusement, playfulness, relaxation, excitement, or (positive) arousal.

Although Zillmann highlighted the excitatory potential of media content that is sought for hedonic reasons, Table 1 shows that aesthetic pleasure can result from pleasing emotions with high or low activation potentials. In contrast to work on narrative formats like film and literature, studies on the experience of nature have highlighted nature’s potential to help people recover from stress and attentional fatigue [21, 149–151]. In contrast to Kaplan [149], who focused on the cognitive benefits of the experience of nature in terms of restoring the capacity for directed attention, Ulrich [21] emphasized the affective benefits of nature experience. For instance, exposure to nature has been linked to reduced fear and physiological arousal as well as more awake relaxation [21, 150]. In addition, van den Berg and colleagues [151] demonstrated that a greater aesthetic preference for natural environments as compared with built cultural habitats is partially explained by the greater affective restoration offered by natural environments.

Nevertheless, nature experiences may also be sought for their activating or energizing potential, as shown by Ryan and colleagues [152] who highlighted the positive effects of the experience of nature for increased subjective vitality. Thus, calmness, peacefulness, serenity, and contentment as well as activation, energy, animation, and excitement are important members of the subclass of pleasing aesthetic emotions.

The subclass of *epistemic* or *knowledge emotions* comprises emotions that have been connected to a search for meaning and insight, such as interest, curiosity, and surprise [5, 10, 153, 154]. Interest and curiosity arise from the novelty and complexity of an aesthetic stimulus and are independent of the pleasingness of the stimulus. Surprise is a more short-lived emotion of neutral valence that serves to orient people to unexpected events [132, 155, 156]. Surprise has been found to intensify other emotions such as interest and amusement as well as confusion and irritation in response to design objects [157].

The notion that aesthetic enjoyment requires cognitive involvement with the stimulus is particularly well represented in measures designed for narrative formats like literature or film. However, the respective intellectual challenges have mostly been considered to be cognitive rather than affective phenomena [86, 87, 158]. In contrast, challenge is among the positive task emotions in [81], and Storm and Storm [159] included *challenged* among the emotion terms related to cognitive states. The latter study categorized being challenged with other emotions that indicate determination and confidence. In light of empirical findings like these and theoretical arguments showing that the feeling of challenge or determination is a positive emotion that is separable from interest and surprise [155, 160, 161], it is reasonable to include intellectual challenge in an emotion measure. While the motivation for understanding has typically been associated with interest [155], we believe that a separate consideration of interest and cognitive challenge could be fruitful in studies of aesthetic emotions. Silvia [153] identified two central appraisals of interest, namely, novelty and comprehensibility. When people feel unable to potentially understand a novel stimulus, their interest fades away. However, interest does not depend on how much cognitive effort will be required to comprehend the stimulus. In contrast, the feeling of intellectual challenge is aroused in situations that present obstacles to understanding, and it is clear that great effort (the prototypical appraisal of challenge [155, 160]) will be required to find meaning in such a stimulus. The resulting feeling of challenge or determination thus might motivate greater efforts toward understanding than interest alone.

Finally, epistemic emotions include not only those linked to searching for meaning, but also emotions resulting from the satisfaction of a drive for sense-making or knowledge (cf. [31, 119, 120]), such as the feeling of insight or knowing [31]. The emotional state of feeling inspired (which is included in some measures in Table 1) is linked to felt insight. Inspiration, and specifically the feeling of being inspired by a stimulus, is triggered by an epistemic event during which “the individual apprehends something ordinarily beyond his or her capacities” ([162], p. 957). In addition to inspiration, gaining insight is pleasurable in itself and increases aesthetic appreciation. A specific type of insight has been described as an *aesthetic aha effect* [143, 144]; it is characterized by a sudden increase in processing fluency, which in turn enhances positive affect and confidence in the truth of one’s insight [163].

#### Negative emotions

Most research has focused on emotions elicited by stimuli that are aesthetically appreciated. However, to study the full range of possible aesthetic emotions, we also need to consider that a specific artwork may not appeal to everyone. Thus, an emotional response can be pure sadness rather than being moved, fear rather than thrill, confusion rather than interest, or boredom rather than enjoyment. As Silvia [132] put it, “Regardless of whether researchers view these feelings as properly aesthetic, people around the world experience these feelings in response to the arts” (p. 48). Indeed, Table 1 contains several negative emotions, ranging from boredom over sadness and disgust to hate.

We sought to limit the range of negative emotions for consideration to those that can occur during typical aesthetic experiences and that conform to our working definition of aesthetic emotions as aesthetically evaluative emotions. We agree with Silvia and colleagues [132, 164] that highly controversial artworks perceived by many to conflict with their key values can lead to feelings of hostility, hatred, contempt, and disgust. Nevertheless, these emotions are unusual aesthetic emotions, considering that recipients normally will not expose themselves to artworks that arouse such intense negative emotions in the first place (or will be likely to discontinue the exposure). The negative emotions selected for our new measure therefore comprise less extreme and more prevalent ones such as anger and irritation rather than hatred and contempt.

We also considered the basic emotions of sadness, fear, and disgust, and we included two negative emotions that frequently occur in response to aesthetically disliked stimuli: confusion and boredom. Confusion is a typical emotion that novices experience when faced with a complex and highly unusual stimulus that they cannot understand [132], that is, when their drive for knowledge is dissatisfied (cf. [120]). Boredom is a response to artworks that strike us as monotonous and lacking interesting aspects as well as variations in affective tonality [5]. Boredom is also the emotion that is most likely to be felt before one’s thoughts begin to wander to other things while dealing with a stimulus; this aspect has been included in some measures developed for film/television or literature (see Table 1).

#### Self-forgetful and self-conscious emotions

Table 1 shows that there is an important difference between measures of aesthetic emotions and experience developed for narrative formats like literature, theater, and film and those developed for non-narrative formats like music and painting. The emotions of empathy, sympathy, and identification play a central role in accounting for the emotional effects of narratives [6, 165–167]. Similarly, self-forgetful states labelled as absorption [168], transportation [169], or flow [170, 171] figure more prominently in such accounts. Overall, this group of emotions includes feeling with and feeling for the characters involved in narratives and the frequently associated experience of losing oneself in the narrative and forgetting time and space.

Therefore, a natural question is whether these emotions should be included in a domain-general measure of aesthetic emotions. As music without words, abstract art, and consumer products do not involve characters with whom recipients can identify or feel empathetic, items assessing identification, empathy, and sympathy such as those that are included in Table 1 are not suited for a domain-general measure. If, however, we conceptualize such feelings at a more abstract level as feelings of affection, attraction, or tenderness, we find related emotional states in measures of, for instance, musical emotions. Thus, we included such emotions among our candidate items for measuring aesthetic emotions.

However, we decided against inclusion of the term *love*. To be sure, several measures listed in Table 1 include love (e.g., [16, 19, 81, 82, 106]). Nevertheless, it is not clear which specific emotion recipients are reporting when they use this term. For instance, many songs, novels, and films express the emotion of romantic love. At the same time, it seems unlikely that recipients who report feeling love while listening, reading, or watching actually feel romantically in love. Although the term *love* can refer to romantic love, it can also refer to liking as well as feelings of tenderness, oneness, or connection. We therefore thought it best to use emotion terms that more clearly distinguish between these possible meanings.

The inclusion of self-forgetful states like absorption, transportation, and flow in a domain-general measure is less debatable when we consider the relevance of these states in response to non-narrative aesthetics. Clearly, it is possible to lose oneself in music, paintings, or landscapes, and not just in narratives. The more important question, to which we will return later, is whether we should consider this experiential state as a specific aesthetic emotion.

Table 1 further offers some insight into which emotions do not need to be included in our new questionnaire. Although self-conscious emotions like pride, shame, guilt, and embarrassment are very important in the general emotion literature [172], they are rarely included in measures of aesthetic emotions. Silvia [132] included pride, shame, and embarrassment in his discussion of unusual aesthetic emotions. However, his examples focus on collective pride in the work of artists with whom recipients identify. While such emotions clearly do occur, they result from self-evaluations rather than evaluations of an external stimulus. That is, they are not elicited by the perceived aesthetic merits of the stimulus, but rather by the implications that these aesthetic merits have for oneself.

Empirical studies on aesthetic emotions that include self-conscious emotions have found that they are reported very infrequently. A study by Juslin and colleagues [73] revealed that the emotions guilt, shame, and humiliation, together with disgust, were the least frequently experienced emotions in response to music. Renaud and Unz [82] excluded shame and guilt in their second study on film and assessed boredom instead. To limit the subclasses of aesthetic emotions to be studied, we did not include items measuring self-conscious emotions, as these are of peripheral importance as responses to perceived aesthetic appeal.

### Studies of the words used to designate dimensions of aesthetic appeal

In addition to considering the measures listed in Table 1, we also looked at empirical studies on words used to describe dimensions of aesthetic appeal to see whether we had missed any potential aesthetic emotions. As the attribution of such dimensions of appeal frequently translates into terms that designate emotional effects (a *moving* film elicits the feeling of *being moved*, etc.), they are candidate items for deriving aesthetic emotions. We considered studies on the aesthetic appeal of visual objects [173, 174], web sites [42], literature [175], and music [176].

Previous research has also identified terms to describe potential elicitors of aesthetic experiences. For instance, collative and affective scales have been used to study the relevant dimensions in the perception of paintings [177] and of architectural environments [178]. Kasmar [179] compiled a list of adjectives characterizing architectural space and used this as a basis to develop the Environment Description Scale (EDS). Craik asked students to describe landscapes (the resulting Landscape Adjective Checklist is included in [20] on pp. 324–325). Lists of *kansei* (the Japanese word for a person’s psychological feelings, impressions, and demands) words are informative with regard to the appeal dimensions of consumer products. *Kansei* engineering (or affective engineering) is a product development methodology that translates customers’ *kansei* into design parameters [18, 180].

Aside from words that are unrelated to emotions or words that designate emotions already presented in Table 1, these studies provided us with a broad sample of frequently used aesthetically evaluative terms. In addition to *beautiful*, these included words like *harmonic*, *rhythmic*, *balanced*, *elegant*, *graceful*, *pretty*, *attractive*, and *mysterious* as positive evaluations and *ugly* and *repulsive* as negative evaluations. Based on these findings, we chose to sample feelings of beauty, harmony, elegance, and perfection and to include the feeling of ugliness among the negative aesthetic emotions.

### Summary

Our project began with the question of which emotions need to be included in a domain-general measure of aesthetic emotions. To answer this question, we started with the Geneva measures of musical emotions (GEMS and GEMIAC; see Table 1). We then extended our search for aesthetic emotions to other art domains beyond music, and finally to aesthetically relevant domains beyond art. Based on a large selection of measures of aesthetic emotions and an integration of theoretical ideas and empirical findings on the range of aesthetic emotions, we arrived at a list of emotions to be included in a preliminary measure of aesthetic emotions. This list includes 24 emotion categories that allow for a highly differentiated characterization of the broader subclasses of aesthetic emotions discussed above. The *prototypical aesthetic emotions* are: (1) feeling of beauty, (2) liking/attraction, (3) captivation, (4) being moved, (5) awe, (6) enchantment/wonder, and (7) nostalgia/longing; the *pleasing emotions* are: (8) joy, (9) humor, (10) vitality/arousal, (11) energy, and (12) relaxation; the *epistemic emotions* are: (13) surprise, (14) interest, (15) intellectual challenge, and (16) insight; the *negative emotions* are: (17) feeling of ugliness, (18) disliking/displeasure, (19) boredom, (20) confusion, (21) anger, (22) uneasiness/fear, and (23) sadness; and the single *self-forgetful emotion* is: (24) flow/absorption. Next, we developed an item set based on the existing items reviewed in Table 1 and also our own expertise and understanding of aesthetic emotions. The German item set used in the present study, along with English translations and our a priori categorization, is presented in S1 Table.

We originally considered developing a questionnaire capturing the whole spectrum of aesthetic emotions that occur in response to the arts (literature, music, visual art, film, etc.) and to aesthetically appealing sights and sounds beyond the traditional arts (advertising, consumer products, natural beauty, etc.). We intended to create a measure that would be encompassing, yet brief enough to be applicable in studies in the field (where aesthetic emotions are typically elicited).

While the item generation was informed by measures of aesthetic emotions across the entire range of aesthetically relevant domains, due to the conflicting constraints of large scope on the one hand and conciseness on the other, we decided to limit our field study to art-elicited emotions. We made this decision for two primary reasons. First, we expected to find a greater range of aesthetic emotions when studying the arts. Natural beauty or consumer products clearly can be as aesthetically appealing as art. However, the resulting experience typically is not as complex and rich with mixed and even negative emotions as the experience of, for instance, tragedies or suspenseful movies. As we wanted to identify the factors underlying aesthetic emotions, it was of great importance to obtain the full range of positive, mixed, and negative aesthetic emotions, which would be accomplished more easily by focusing on the arts.

Second, we wanted to select emotions for a measure of aesthetic emotions rather than emotions in general. As can be seen in Table 1, measures of emotional responses to advertising or consumer products often include emotions like envy, shame, pride, hope/optimism, and respect. These are atypical as art-elicited emotions and likely represent utilitarian emotions (e.g., envy of others who own a product). When studies move beyond the traditional arts, the reported emotional responses usually represent mixtures of aesthetic and utilitarian emotions. It may be difficult to separate these kinds of emotions in a factor analysis, which is why we limited the empirical basis for item selection to the arts.

## Method

We report how we determined our sample size, all data exclusions (if any), all manipulations (if any), and all measures in the study. The raw data, analysis scripts and outputs, and study materials are available at Open Science Framework (https://osf.io/q8zv5; doi:10.17605/OSF.IO/Q8ZV5).

We developed our new measure of aesthetic emotions, the Aesthetic Emotions Scale (Aesthemos), by combining a top-down theoretical approach with a bottom-up empirical approach, which led to 24 emotion categories to be studied. We compiled an item set representing these categories. Subsequently, we conducted a field study to select the best items for inclusion in the final scale.

### Ethics statement

The study was conducted in full accordance with the World Medical Association’s Declaration of Helsinki and the Ethical Guidelines of the German Association of Psychologists (DGPs). Formal ethics approvals for the type of research reported in this paper are not required by these guidelines or by German laws. Moreover, by the time the data were acquired (2014), it was also customary at Freie Universität Berlin and at most other German universities not to seek ethics approvals for simple behavioral studies. The authors evaluated this study as not creating any harm or distress to the participants. Under this assumption—which, according to German law, is at the full discretion of the authors and for which they hence assume full responsibility—and in line with the above-mentioned rules and customary procedures, a formal ethics approval or waiver of such an approval was not required, and hence we did not request these.

The participants were explicitly informed about the task they were expected to perform, the anonymity of the data obtained through this task, the fully voluntary nature of their participation, and their right to withdraw from the study at any time, and thereafter they gave their informed consent in writing. The consent forms were separated from the completed questionnaires, so that it is impossible to rematch consent forms and questionnaires. Thus, the questionnaires and stored data are completely anonymous.

### Item generation

Our initial set contained 122 emotion items. As these were too many to be tested in a field study, we conducted an initial online study to narrow down the number of items to be included. We emailed a study invitation to all people who had signed up for the participant newsletter of the Cluster “Languages of Emotion” at Freie Universität Berlin and to personal acquaintances. We received responses from 77 participants (57.1% women, 29.9% men, 13.0% did not report their gender; age range 22–75 years, *M* = 38.1, *SD* = 13.0, 13.0% did not report their age) who rated each item with regard to how frequently they would use it to describe their emotional reaction during an aesthetic experience (1 *never* to 5 *very frequently*). Based on these ratings, we eliminated those items within the emotion categories that the participants would rarely use to describe their aesthetic emotions. We further engaged in a final discussion of all remaining candidate items and decided to eliminate emotions that were not likely to be directed toward a piece of art, music, or design but rather toward its creator or user. Most notably, this led to the exclusion of admiration, which typically is directed toward the artist’s or designer’s talent or artisanship rather than the produced object (for supporting evidence, see Desmet [16], who found designers or users of consumer products rather than the products per se to be the objects of admiration). The resulting reduced item set contained 75 items (see overview in S1 Table for the full item set and our a priori categorization), and these were included in the field study.

### Participants and procedure

The field study was conducted between May and August of 2014. We sampled a broad range of events of aesthetic interest, such as concerts, musicals and dance theaters, theatrical performances, readings, museum exhibitions, and film screenings. We began the study with the goal of recruiting 500 participants who attended one of at least 20 different events (which is the absolute minimum number of events required for using the complex option in Mplus). After obtaining the event organizers’ consent, our research assistants approached audience members aged 18 and older when an event was over and invited them to participate in the study. Participants filled in a questionnaire, which typically took between 15 and 20 minutes to be completed, and received 5 Euro as a compensation for their effort.

Data were collected during 27 events with the aim of obtaining at least five valid responses per event. We received questionnaires from 507 respondents. Four participants only answered some initial questions and left the rest of the questionnaire blank. We further excluded from the data analysis six participants who did not sincerely answer the items (e.g., they provided the same rating for all items or for all items on a page), along with two events for each of which we had obtained only one valid questionnaire. Finally, one questionnaire was not analyzed because the participant reported being younger than 18 years old. The resulting final sample includes 494 participants who attended one of 25 events (between 6 and 38 participants per event; see S2 Table for a list of the specific events and *n* per event). The participants reportedly attended the respective event for between 10 and 360 minutes, *M* = 119.1 minutes, *SD* = 43.0.

The participants (60.5% women, 39.3% men, 0.2% other gender) included in the analyses were between 18 and 86 years of age, *M* = 40.6, *SD* = 16.8 (3.2% did not report their age). All participants were fluent in German, and 88.1% reported German as their native language. On average, the sample was highly educated, with 87.4% having qualified for college entrance and 59.5% having graduated from college or university. Concerning activities and hobbies relevant to arts and aesthetics, 39.1% reported themselves to be (lay) actors/actresses, artists, musicians, writers, or photographers, or to be studying/having studied art, music, or literature.

### Measures

The questionnaire was presented in German. After providing demographic information, participants answered questions concerning the event they had visited and the aesthetic emotions they experienced. They rated 75 emotion items in terms of the emotional effect the event had on them on a 5-point scale ranging from 1 (*never*) to 5 (*very frequently*).

Participants also reported on how frequently they performed a range of activities that are likely to involve aesthetic emotions (e.g., listening to music, singing, painting, reading, and going to the theater). Finally, participants rated their general mood and their need for affect, and they were provided with some additional space to comment on the study. As these additional data were not needed for the scale development, we do not report them here.

### Data analysis

We analyzed the dimensional structure of the items in several steps. First, we conducted a confirmatory factor analysis (CFA) to test whether the 75 items followed the theoretically expected structure of 24 dimensions, using the software Mplus 7.4 [181]. The estimation procedure did not converge properly, due to a high multicollinearity of the latent factors. For this reason, we explored the dimensionality of the item set. We conducted an exploratory factor analysis (EFA) for ordinal response variables using the WLSMV estimator in Mplus with the analysis option TYPE = COMPLEX MISSING, to take into account that participants were nested within the 25 events. Overall, only 719 out of 37,050 (1.9%) possible responses were missing. A priori, 24 factors were specified and an oblimin rotation between the factors was performed. We carefully checked whether the loading structure was in line with our theoretical expectations and interpreted the correlations between the factors.

Second, because of the high correlations between the factors, we explored whether it is possible to reduce the number of factors. We conducted a parallel analysis based on the polychoric correlation matrix of the items using the function fa.parallel() in the R-package psych [182]. Based on an examination of the scree plot and the parallel analysis, we conducted a second EFA with seven oblimin-rotated factors. In addition, we checked the stability of our factor solution by means of various supplementary sensitivity analyses, such as principal component analyses and EFAs for continuous data. Both types of analyses supported our solution (detailed analyses can be provided on request).

Third, considering the two EFAs with 24 and seven extracted factors, respectively, we selected a subset of items to represent different scales. The selection process was guided by psychometric indices, such as factor loadings, as well as theoretical considerations. Finally, we computed the item statistics and reliabilities of the scales and examined their correlations.

## Results

Table 2 shows the oblimin-rotated loading matrix (pattern matrix) obtained by the EFA using 24 factors (the factor structure maxtrix is available as S3 Table and the factor correlations are presented in S4 Table). The aim of the analysis was to check whether the loading structure supported our a priori classification of emotion items. The model fit was excellent, χ^2^ (1251) = 1291.04, *p* = .21, *CFI* = 1.00, *TLI* = 1.00, *RMSEA* = 0.01. Although the loading structure was closely in line with a simple structure, the loading matrix did not entirely follow the structure we initially assumed.

**Table 2:** Oblimin-rotated loadings of an exploratory factor analysis with 24 factors.

	Factor																							
Item (subscale number)	1	2	3	4	5	6	7	8	9	10	11	12	13	14	15	16	17	18	19	20	21	22	23	24
**3 Invigorated me (9)**	**.77**	-.00	-.01	.03	-.01	.01	.00	.04	.01	.12	.04	.00	.01	-.05	.13	.07	-.03	-.01	.00	-.09	-.03	.02	-.03	.08
**2 Spurred me on (9)**	**.65**	-.10	-.01	-.06	.02	.04	.06	.03	.02	-.01	.07	.07	-.04	.04	.06	-.01	.00	.02	-.02	.20	-.05	-.10	.03	-.07
1 Filled me with longing	.29	.05	.00	-.01	.04	.07	.01	-.02	.05	-.03	.08	-.04	.27	-.06	.07	.02	.02	.08	-.03	.23	.18	-.19	.26	-.27
22 Felt depressed	-.16	**.57**	.02	.05	.09	.00	.19	.09	-.09	.04	.00	.06	.04	.12	.01	-.06	-.07	.04	.05	-.02	-.11	.01	.09	-.01
**5 Felt oppressive (20)**	.06	**.46**	.09	.04	.05	-.06	.08	-.06	.06	-.03	.08	-.03	-.16	.23	-.13	-.11	-.09	.10	.08	-.07	.01	.00	.16	.08
21 Made me feel uncomfortable	-.13	.39	.01	-.21	.27	.03	.13	.09	-.09	.10	-.09	.01	.13	.09	.05	-.01	.00	-.07	.13	-.04	.06	.05	-.07	-.01
**16 Worried me (20)**	-.08	.30	.16	.20	.10	-.01	.09	-.16	-.09	.11	-.02	.00	-.08	.22	.04	-.09	.10	.04	.13	-.02	-.05	-.08	.25	-.08
**17 Challenged me intellectually (14)**	-.02	.04	**.78**	.03	.06	-.04	.10	-.05	-.02	.03	.04	-.01	-.06	-.01	.09	-.01	.00	.10	.06	.01	.02	-.06	-.02	-.02
**24 Was mentally engaged (14)**	.00	-.02	**.76**	.00	-.08	.07	.02	.10	.03	.03	.01	.07	.13	.03	-.08	-.01	-.05	.03	.07	-.05	-.01	.02	-.02	.08
**23 Felt a sudden insight (15)**	-.08	.06	**.33**	-.05	.01	.01	.13	-.06	.00	.05	-.10	.27	.02	.05	.12	.11	.03	.09	-.17	.18	-.02	.15	.13	.13
**15 Felt deeply moved (3)**	-.02	.04	.05	**.62**	.05	.09	-.07	.11	.14	.01	.06	.09	.07	-.07	.08	.00	-.09	.00	.02	.06	-.01	-.01	.09	-.01
49 Was overwhelmed	-.04	-.03	-.01	**.37**	.12	-.05	-.04	.09	.04	.24	.22	.04	.04	.01	.08	-.04	.06	.05	.06	**.35**	-.03	-.03	-.05	.09
6 Gripped me	.19	.03	.12	**.32**	.00	.09	.00	.05	.18	.11	.02	-.05	-.07	.05	.03	-.02	.08	.02	.05	.00	-.15	.04	.03	.20
62 Felt that time was flying	.15	.01	-.06	.29	-.08	.06	.16	.12	-.05	.05	-.08	-.17	.06	.08	.11	.04	.04	.13	-.12	-.09	-.19	-.02	-.02	.21
**8 Made me angry (19)**	-.02	.04	-.03	.02	**.72**	-.04	.06	-.02	-.02	.06	-.02	.02	-.05	.05	-.06	.02	-.07	.00	-.06	-.02	.08	.02	.12	-.07
**35 Made me aggressive (19)**	.04	.04	.00	.06	**.62**	.01	.08	-.05	-.02	-.13	-.13	.11	-.07	.08	.01	-.03	-.06	.09	.14	.03	.01	.09	-.01	.03
36 Disliked it	-.09	.10	.03	-.11	**.40**	.09	.04	-.03	-.17	.08	.01	-.04	.15	.14	.02	-.07	-.08	-.18	.08	-.08	.23	.10	-.16	.08
56 Scared me	-.03	.05	-.03	.21	**.31**	-.11	.01	-.04	-.05	.10	-.05	-.01	.08	.15	.02	-.06	-.02	.07	.32	-.12	-.15	.07	.18	.03
**12 Touched me (3)**	.10	.06	.11	.25	.14	**.36**	-.10	.11	.18	.00	.02	.08	.08	-.08	-.03	-.01	.00	-.03	-.02	.01	-.10	-.01	.19	.18
**13 Delighted me (7)**	.22	-.13	.03	.06	-.07	.37	.03	.15	-.01	.00	-.07	.04	.03	-.10	-.03	.25	.24	-.01	-.08	.01	-.04	.02	-.14	-.05
18 Was not aware of myself	.03	.04	.07	-.06	.03	-.01	**.78**	.00	.01	.04	.03	.06	.01	-.05	.03	.02	.04	-.07	.00	.01	.04	.00	.02	-.01
**19 Was impressed (2)**	.13	.06	.05	.06	-.08	.00	.12	**.40**	.22	.07	.11	.07	-.05	-.11	-.04	-.09	.02	.15	.06	.02	-.07	.07	.08	.10
14 I found it perfect	.11	.18	.01	.23	-.09	.12	-.06	.46	-.02	.08	.00	.17	.05	.04	.11	-.02	.10	.08	-.11	.05	.11	.00	.01	-.10
**28 I found it beautiful (1)**	-.01	-.12	-.09	.02	-.05	.11	.00	.04	**.53**	-.03	.14	.05	.10	.05	-.02	.08	.18	.06	.02	.00	.05	-.20	-.04	-.02
29 Was attracted	.10	-.05	.06	.10	-.06	.01	-.01	-.04	**.49**	.11	-.05	.14	.08	.00	.12	-.05	.02	.07	-.02	.02	.00	-.08	-.07	.06
**25 Liked it (1)**	.00	.02	.04	.12	-.04	.07	.00	.12	**.48**	.11	.09	-.18	.02	-.19	.08	.10	.02	.15	-.07	-.01	-.13	.05	.11	.01
30 Made me feel enthusiastic	.12	.00	.05	.07	-.04	-.02	-.04	.14	**.48**	.18	-.13	.04	.02	.05	.09	.11	.06	-.07	-.07	.21	-.06	.05	-.02	.08
**44 Surprised me (12)**	.05	.04	.03	-.02	-.02	.11	.05	-.01	.03	**.70**	.02	-.04	.00	.02	.04	.14	-.03	.08	.05	-.05	-.02	-.01	-.05	.03
**31 Baffled me (12)**	.13	.03	.04	.09	.01	-.10	.07	.09	.10	**.66**	-.04	.03	.08	-.04	-.09	-.04	.05	.03	.08	.12	-.03	.05	-.03	-.01
57 Astonished me	.03	-.30	.14	-.05	.10	-.03	.04	.33	-.01	**.41**	.18	.02	-.02	.09	-.03	-.01	.02	.11	.09	.05	-.14	-.03	.11	-.02
**9 I found it sublime (4)**	.11	-.02	.19	.06	-.08	-.02	.13	.05	.03	-.05	**.63**	.13	.01	.00	.02	-.01	.04	-.08	-.10	.04	.05	.19	-.02	-.08
48 I found it graceful	.03	.04	-.13	.01	-.10	.04	-.04	-.02	-.03	.08	**.53**	.14	.18	.08	.04	.04	-.03	.09	-.02	.10	.08	-.10	-.04	.09
72 I found it harmonious	.10	.08	-.05	-.01	-.02	-.07	.05	.07	.04	.04	**.33**	.00	.00	-.09	.09	.16	.25	.05	-.01	-.05	.03	-.30	-.07	.25
32 Felt humbled	.03	.04	.07	.01	.06	-.05	.07	.05	-.01	-.07	.06	**.76**	.02	-.04	.02	-.02	.07	.07	.03	.00	-.10	.03	.04	-.03
**51 Felt awe (4)**	.02	-.12	-.13	.04	.00	.18	.17	.02	.07	.12	.24	**.51**	.07	.04	.05	-.15	-.11	.02	.13	-.03	.07	-.14	.01	.06
**45 Sensed a deeper meaning (15)**	.01	.05	.21	.16	-.02	.20	.02	-.14	.05	.25	-.05	.27	.06	.19	.04	-.01	.04	.10	-.25	-.02	.02	.00	.11	.10
**41 Made me feel sentimental (6)**	.00	.00	.01	.02	-.03	.02	.00	.01	.04	.00	.02	-.02	**.85**	-.02	.03	.01	.00	-.05	.02	.01	-.04	.06	.08	.06
**33 Made me feel nostalgic (6)**	-.04	.00	.10	.01	.00	-.04	.07	-.01	-.03	.06	.05	.10	**.64**	.05	-.02	.08	.06	.11	-.07	.01	.09	-.14	.01	-.15
27 Repelled me	-.05	.15	.03	-.10	.16	-.01	.05	.03	-.03	-.03	.05	-.03	.01	**.62**	-.02	.09	.03	-.14	.10	.03	.04	.14	.01	.01
26 Was shocking to me	-.07	.08	.06	.18	.12	-.06	.06	.01	.15	.18	-.02	-.01	-.02	**.37**	-.02	.03	-.13	.11	.12	-.11	-.05	.16	.14	-.06
**39 Energized me (10)**	.11	.04	.05	.04	-.03	-.04	.05	.07	.08	-.09	.05	.05	.03	-.04	**.74**	-.01	-.01	-.04	.02	-.01	-.06	.06	-.03	-.02
52 Perked me up	.04	-.08	-.15	.14	-.07	.16	.04	-.05	-.05	.13	.06	-.06	-.03	-.02	**.45**	.22	.05	.05	.12	-.01	-.09	-.07	-.08	.10
**53 Motivated me to act (10)**	.16	-.07	-.07	-.04	.12	.09	.03	-.09	-.07	.09	-.07	.06	.03	.02	**.44**	-.04	.13	.20	-.05	.17	.11	-.08	.06	.05
54 Felt absorbed in the experience	.11	-.20	.11	.04	-.03	.01	.23	-.07	-.01	-.02	-.07	-.02	.24	.20	**.34**	-.01	.00	.15	-.07	.12	-.04	-.02	.05	.02
42 Inspired me	.24	.01	.16	-.18	-.04	.01	-.14	.05	.10	.04	.00	.17	.11	.07	**.32**	.02	-.02	.24	-.04	.10	.12	.02	.10	.09
**59 Was funny to me (8)**	.04	-.02	-.05	.05	.00	-.02	.05	.00	-.03	.01	-.04	-.01	.02	.05	-.10	**.93**	-.05	.04	-.03	-.03	.04	.08	.02	.07
**71 Amused me (8)**	-.05	.00	.07	-.05	.00	-.01	-.03	.01	.04	.00	.05	-.03	.02	.00	.09	**.90**	.00	-.05	.06	.04	-.05	-.06	.02	-.07
43 Made me merry	.10	-.01	-.05	-.07	.02	.20	-.06	-.08	.08	.11	.03	.04	.03	-.11	.13	**.54**	.10	.07	.02	.05	-.05	-.01	-.09	-.04
4 Made me cheerful	.27	-.06	-.06	.03	-.05	.21	-.04	.02	.11	.02	-.03	-.05	.09	-.11	.01	**.35**	.18	.02	.02	.03	.03	.01	-.15	-.06
**7 Calmed me (11)**	-.09	-.05	-.01	-.09	-.06	.00	.08	.00	.04	.04	.06	.09	.10	-.06	.01	-.13	**.63**	.02	-.06	.06	-.01	.08	.04	-.06
**74 Relaxed me (11)**	.01	-.04	-.17	.07	-.16	-.01	.03	.05	.08	.07	-.06	.11	.03	.01	.07	.14	**.51**	-.06	-.08	-.04	.20	-.09	-.03	.22
10 Made me feel content	.17	-.01	.05	-.02	-.14	.13	-.06	.08	.13	-.11	.08	-.03	.06	.11	.04	.01	**.44**	.02	.02	.11	-.16	.05	-.18	-.05
**11 Made me happy (7)**	.19	.01	.03	.05	-.06	.25	-.01	.06	.10	-.06	.09	-.07	.05	.05	-.01	.12	**.36**	.02	.02	.21	-.11	-.03	-.25	-.04
55 I found it pleasant	.03	-.21	-.06	-.13	-.09	.06	.07	.17	.20	-.07	.10	-.13	.06	-.01	.13	.11	**.35**	.23	-.04	-.10	-.04	-.11	.07	-.05
**34 Made me curious (13)**	.02	.04	.17	-.01	.05	-.05	-.09	.06	-.02	.07	-.04	.12	.03	-.10	-.02	.02	.00	**.67**	.04	.03	.02	-.02	-.04	.05
**46 Sparked my interest (13)**	-.10	-.01	.07	.06	.02	.18	.06	.05	.24	.14	.08	.04	-.09	.06	.11	.00	-.02	**.47**	-.10	-.03	-.09	.02	.01	.09
**61 Felt confused (18)**	.01	.00	.11	.06	.08	-.08	.11	-.01	.01	.06	-.05	.04	.02	.08	-.02	.07	-.03	-.01	**.72**	.00	.07	.01	-.01	.03
**69 Was unsettling to me (18)**	-.09	.16	.07	.02	-.10	.24	.09	-.03	-.05	.12	-.06	.00	-.10	.05	.01	-.09	-.09	-.03	**.56**	.03	.09	.10	.25	-.11
40 Irritated me	-.01	.09	.12	-.14	.11	.00	.03	-.02	-.10	.09	.03	.10	.04	.06	.02	.06	-.02	.03	**.49**	-.07	.04	.15	-.05	.01
60 I found it unpleasant	-.10	.10	-.07	-.10	.16	-.14	-.06	-.02	-.08	.02	.00	.02	.09	.14	.07	-.02	.02	-.01	**.44**	-.02	-.03	.28	-.01	.00
**50 Was enchanted (5)**	.10	-.04	-.05	.05	-.03	.04	.09	.06	.08	.15	.15	.06	.13	-.01	.02	.07	.05	.03	-.01	**.48**	-.04	-.04	-.05	.01
**37 Felt something wonderful (5)**	.09	-.03	.02	.06	-.02	.12	.12	.14	.13	-.09	.03	.02	.13	-.02	.16	.05	.00	.12	-.08	**.39**	.04	.00	-.17	-.04
70 Put me in a dreamy mood	.02	-.06	-.01	-.05	-.17	-.01	.06	-.07	-.03	-.04	.07	.03	.29	-.14	.08	.15	.17	-.03	.12	**.36**	.11	-.07	.08	.13
65 Was enraptured	.02	-.10	-.07	.22	-.08	.06	.02	.20	-.02	.14	.10	-.01	.01	.11	.17	.07	-.05	.01	-.04	**.34**	-.06	.02	.09	.12
**64 Felt indifferent (17)**	-.09	-.01	-.05	.01	.03	-.10	.20	.07	-.07	-.09	-.04	-.08	.01	.16	-.04	-.01	-.05	-.03	.10	.00	**.59**	.00	-.10	-.13
**68 Bored me (17)**	-.07	-.01	-.03	-.14	.14	-.03	.03	-.15	.00	-.02	.14	-.11	-.01	-.12	-.10	-.05	-.02	-.04	.05	.05	**.57**	.28	.02	.02
38 Tired me	-.04	-.07	.15	-.06	.16	.01	.01	-.08	-.17	-.11	.15	.00	.10	.04	-.06	-.11	.15	-.03	.11	-.10	**.49**	.06	-.01	.02
**58 I found it ugly (16)**	-.09	.01	.04	.00	.19	-.03	.05	.00	-.05	.06	.08	.01	-.06	.09	.05	.01	.05	-.12	.08	-.05	.07	**.63**	.02	-.01
**63 I found it distasteful (16)**	.01	.06	-.22	.01	-.01	.01	.14	-.02	-.09	-.04	.00	-.05	.01	.23	-.07	.12	-.04	.13	.17	-.02	.20	**.51**	-.04	-.06
**67 Made me sad (21)**	.01	.04	.02	.04	.09	.03	.09	.04	.01	-.07	-.04	.07	.17	.11	-.02	.01	-.08	-.01	.06	-.05	-.03	.02	**.67**	.08
**20 Made me feel melancholic (21)**	-.07	.17	-.08	.13	.07	-.06	.08	.00	-.02	.02	-.03	.13	.23	-.15	-.03	.02	.09	.04	.04	.06	-.05	-.01	**.50**	.03
73 Stimulated my thoughts	.08	.01	.22	.00	-.08	.02	.00	-.04	.03	.01	.02	.03	.03	-.04	.04	.00	.02	.21	-.04	.04	.00	-.05	.16	**.52**
**75 Fascinated me (2)**	-.01	-.04	.05	.03	-.01	.03	.03	**.32**	.17	.15	-.03	.10	-.06	.07	.10	.05	.02	.12	.02	.18	.06	-.11	.00	**.33**
66 Moved me	-.03	.03	.03	.02	.02	.26	.03	.17	.08	.01	.05	-.01	.01	.05	.14	.02	-.12	.02	.01	.19	-.21	-.10	.24	.30
47 Agitated me	.05	.18	-.02	.17	.07	.14	.18	-.09	.04	-.08	.03	.06	.13	.07	-.04	-.10	-.11	.17	.21	.15	-.21	.09	.00	.26

*Note*. Factors, loadings, and item assignments to factors that were predicted a priori are marked in gray. The 42 items included in the final Aesthemos along with the respective subscale number and loadings with *p* < .05 and and λ ≥ |.30| are highlighted in bold. Note that some loadings with λ ≥ |.30| are not significant at *p* < .05 due to large standard errors.

In Table 2, we have marked the factors and item assignments to factors that matched our predictions in gray. Examining the loading matrix, 20 out of the 24 factors were interpretable in terms of our a priori categories: vitality/arousal (F24_1), uneasiness/fear (F24_2), intellectual challenge (F24_3), being moved (F24_4), anger (F24_5), flow/absorption (F24_7), captivation (F24_8), feeling of beauty (F24_9), surprise (F24_10), awe (F24_12), nostalgia/longing (F24_13), energy (F24_15), humor (F24_16), relaxation (F24_17), interest (F24_18), confusion (F24_19), enchantment/wonder (F24_20), boredom (F24_21), feeling of ugliness (F24_22), and sadness (F24_23). Moreover, 40 out of the 75 items loaded on the predicted factor. For ten factors, two items each loaded according to our a priori categorization, while for five factors (F24_10, F24_16, F24_17, F24_20, and F24_21), all three items loaded as expected.

In contrast, items intended to measure liking/attraction, joy, insight, and disliking/displeasure did not form the respective factors that we had expected a priori. Liking/attraction items loaded on the feeling of beauty factor (F24_9), and insight items tended to load on the intellectual challenge factor (F24_3). The joy items and disliking/displeasure items did not show a clear loading pattern. In addition, there were five factors (F24_1, F24_4, F24_7, F24_8, and F24_9) on which only one item loaded as predicted, which means that items from another a priori category would be needed to form a scale with at least two items.

To assess the number of factors that could represent the item correlation matrix most parsimoniously and to check whether the partially inconsistent loading structure might be due to overfactorization, we scrutinized the eigenvalues of the polychoric correlation matrix and performed a parallel analysis. The parallel analysis and examination of the scree plot (shown in S1 Fig) suggest that seven to eight factors suffice to explain the matrix most parsimoniously.

Table 3 shows the oblimin-rotated loadings (pattern matrix) of the items on seven factors (the factor structure matrix and factor correlations are reported in S5 and S6 Tables, respectively). The model fit was acceptable, χ^2^ (2271) = 2623.74, *p* < .05, *CFI* = 0.97, *TLI* = 0.96, *RMSEA* = 0.02. The factors can be interpreted in the following way: negative emotions (F7_1), prototypical aesthetic emotions (F7_2), epistemic emotions (F7_3), animation (F7_4), nostalgia/relaxation (F7_5), sadness (F7_6), and amusement (F7_7).

**Table 3:** Oblimin-rotated loadings of an exploratory factor analysis with seven factors.

Factor	1	2	3	4	5	6	7	Subscale (number)
*Factor 1*: *Negative emotions*								
**63 I found it distasteful**	**.84**	.04	-.18	-.04	.05	-.12	.15	Feeling of ugliness (16)
**58 I found it ugly**	**.79**	.09	-.05	-.10	-.03	-.13	-.02	Feeling of ugliness (16)
27 Repelled me	**.82**	-.01	-.01	.01	.00	.04	.06	--
60 I found it unpleasant	**.79**	-.04	-.05	-.08	.01	.05	.03	--
36 Disliked it	**.76**	-.20	-.05	.00	.08	-.05	-.10	--
21 Made me feel uncomfortable	**.70**	-.12	.06	-.04	.06	.14	.00	--
**61 Felt confused**	**.77**	.13	.15	-.05	.01	.07	.13	Confusion (18)
**69 Was unsettling to me**	**.66**	.13	.11	-.14	-.05	.22	-.02	Confusion (18)
26 Was shocking to me	**.58**	.27	.19	-.11	-.24	.25	.04	--
40 Irritated me	**.74**	-.01	.22	-.03	.04	-.06	.08	--
**35 Made me aggressive**	**.67**	-.13	.03	.24	-.21	.14	-.17	Anger (19)
**8 Made me angry**	**.60**	-.11	-.03	.06	-.13	.16	-.16	Anger (19)
**64 Felt indifferent**	**.57**	-.16	-.14	-.09	.31	-.23	-.09	Boredom (17)
**68 Bored me**	**.49**	-.18	-.13	-.19	.35	-.25	-.13	Boredom (17)
38 Tired me	**.44**	-.23	.06	-.10	**.45**	-.23	-.20	--
**16 Worried me**	**.43**	.05	.23	-.08	-.10	**.40**	-.12	Uneasiness (20)
**5 Felt oppressive**	**.39**	.10	.22	-.19	-.18	.27	-.21	Uneasiness (20)
56 Scared me	**.60**	.11	.05	-.03	-.21	**.38**	-.03	--
22 Felt depressed	**.50**	.09	.16	-.13	-.06	**.42**	-.16	--
18 Was not aware of myself	**.42**	.05	.20	.18	.25	.03	-.04	--
55 I found it pleasant	**-.42**	.19	.12	.07	.27	-.15	**.33**	--
*Factor 2*: *Prototypical aesthetic emotions*								
**15 Felt deeply moved**	-.10	**.58**	-.05	.11	-.08	**.42**	-.11	Being moved (3)
**12 Touched me**	-.21	**.43**	.10	.11	-.07	**.42**	.01	Being moved (3)
**31 Baffled me**	.26	**.57**	.28	.00	-.04	-.04	.17	Surprise (12)
**44 Surprised me**	**.30**	**.41**	**.32**	.05	-.15	-.10	**.36**	Surprise (12)
57 Astonished me	.13	**.53**	**.35**	.06	-.01	-.12	.06	--
**19 Was impressed**	-.12	**.64**	.26	.03	.00	.02	-.06	Fascination (2)
**75 Fascinated me**	-.08	**.47**	.28	.24	-.04	.07	.10	Fascination (2)
49 Was overwhelmed	.14	**.69**	-.03	.24	.09	.15	-.07	--
6 Gripped me	-.07	**.49**	.14	.16	-.24	.16	.08	--
30 Made me feel enthusiastic	-.20	**.50**	.12	.19	-.06	.05	.25	--
**25 Liked it**	**-.40**	**.51**	.16	-.05	-.08	.13	.29	Feeling of beauty/liking (1)
**28 I found it beautiful**	**-.39**	**.43**	.06	-.01	.28	-.03	.22	Feeling of beauty/liking (1)
29 Was attracted	**-.31**	**.36**	.25	.18	.04	.05	.09	--
65 Was enraptured	.01	**.51**	-.09	.38	-.01	.17	.06	--
72 I found it harmonious	-.28	.25	.10	.14	.25	-.08	.18	--
14 I found it perfect	-.09	**.51**	.09	.21	.09	.09	-.03	--
**9 I found it sublime**	.08	**.44**	.11	.12	**.41**	-.25	-.24	Awe (4)
**51 Felt awe**	.10	**.41**	.16	.26	**.32**	.08	**-.34**	Awe (4)
10 Made me feel content	-.20	**.36**	-.02	.17	.20	-.28	.24	--
*Factor 3*: *Epistemic emotions*								
**17 Challenged me intellectually**	.08	-.14	**.86**	.02	.00	-.10	-.10	Intellectual challenge (14)
**24 Was mentally engaged**	-.01	.03	**.80**	-.10	.08	-.04	-.04	Intellectual challenge (14)
73 Stimulated my thoughts	-.24	.04	**.47**	.18	-.03	.24	.06	--
**34 Made me curious**	-.12	.04	**.56**	.13	-.03	.04	.05	Interest (13)
**46 Sparked my interest**	-.14	**.33**	**.45**	.15	-.15	.09	.10	Interest (13)
**23 Felt a sudden insight**	.08	-.08	**.53**	.21	.11	.19	-.01	Insight (15)
**45 Sensed a deeper meaning**	-.05	.18	**.46**	.15	.03	.24	-.02	Insight (15)
*Factor 4*: *Animation*								
**53 Motivated me to act**	.04	-.11	.11	**.72**	.08	.05	.05	Energy (10)
**39 Energized me**	.01	-.02	.07	**.68**	-.03	.03	.03	Energy (10)
**2 Spurred me on**	-.11	.12	.00	**.71**	.01	-.20	-.02	Vitality (9)
**3 Invigorated me**	-.06	.16	.03	**.63**	-.14	-.19	.16	Vitality (9)
52 Perked me up	.01	.13	-.07	**.49**	-.11	.02	**.37**	--
42 Inspired me	-.10	-.06	**.42**	**.55**	.11	.02	.07	--
**37 Felt something wonderful**	-.09	.28	-.02	**.51**	.22	-.03	.07	Enchantment (5)
**50 Was enchanted**	.00	**.46**	-.06	**.40**	.29	.03	.06	Enchantment (5)
54 Felt absorbed in the experience	.06	-.11	.21	**.60**	.15	.15	.10	--
62 Felt that time was flying	-.13	.24	.02	.25	-.23	.21	.21	--
*Factor 5*: *Nostalgia/relaxation*								
**33 Made me feel nostalgic**	-.01	-.06	.18	.06	**.67**	.29	.17	Nostalgia (6)
**41 Made me feel sentimental**	.00	.00	.00	.09	**.60**	.47	.20	Nostalgia (6)
1 Filled me with longing	-.10	.00	-.04	**.38**	**.42**	.20	.03	--
70 Put me in a dreamy mood	-.11	.01	-.05	.28	**.53**	.13	.20	--
48 I found it graceful	.02	**.40**	-.02	.20	**.43**	-.03	-.08	--
**7 Calmed me**	-.18	.14	.10	-.05	**.49**	-.13	.06	Relaxation (11)
**74 Relaxed me**	-.27	.20	-.02	.03	**.33**	-.10	**.30**	Relaxation (11)
*Factor 6*: *Sadness*								
**67 Made me sad**	.17	-.02	.12	-.03	.13	**.74**	-.06	Sadness (21)
**20 Made me feel melancholic**	.02	.08	.04	-.07	.27	**.70**	-.03	Sadness (21)
66 Moved me	-.13	**.31**	.13	**.30**	-.13	**.42**	.06	--
47 Agitated me	**.30**	.23	.12	.23	-.08	**.41**	-.09	--
*Factor 7*: *Amusement*								
**71 Amused me**	.08	-.09	.00	.02	.05	.03	**.92**	Humor (8)
**59 Was funny to me**	.18	-.09	-.04	-.04	.00	.06	**.91**	Humor (8)
43 Made me merry	-.06	.04	.02	.27	.06	-.09	**.65**	**--**
**13 Delighted me**	-.24	.24	-.05	.25	.09	-.23	**.42**	Joy (7)
**11 Made me happy**	-.15	**.40**	-.09	.27	.19	-.28	**.33**	Joy (7)
4 Made me cheerful	-.16	.15	-.12	.25	.11	-.21	**.55**	--
32 Felt humbled	.02	.16	**.35**	.21	.29	.14	**-.36**	--

*Note*. The 42 items included in the final Aesthemos and loadings with *p* < .05 and λ ≥ |.30| are highlighted in bold. Note that some loadings with λ ≥ |.30| are not significant at *p* < .05 due to large standard errors.

Based on the loading structures of both EFAs, we selected 42 items and grouped them into 21 scales with two items each (see Tables 2 and 3) to form our new measure, the Aesthetic Emotions Scale (Aesthemos; the questionnaire is included in S1 Appendix). Basic criteria were a) a common loading on one of the factors of the EFA with 24 factors and b) a similar loading profile in the loading matrix of the EFA with seven factors. These criteria were met by the items selected for 17 of the 21 scales: (1) feeling of beauty/liking, (2) fascination, (5) enchantment, (6) nostalgia, (8) humor, (9) vitality, (10) energy, (11) relaxation, (12) surprise, (13) interest, (14) intellectual challenge, (16) feeling of ugliness, (17) boredom, (18) confusion, (19) anger, (20) uneasiness, and (21) sadness.

Theoretical considerations and previous empirical findings guided our selection of the remaining eight items and four scales that were not as clearly supported by the present analyses: (3) being moved, (4) awe, (7) joy, and (15) insight. In particular, we considered a study [183] on the conceptual space of the aesthetic emotion terms that were also included in the present study.

Although being emotionally moved loaded on two factors in our EFA with 24 factors, the study by Hosoya and colleagues [183] as well as studies on being moved [122, 123] have shown that feeling deeply moved and feeling touched are closely related. As the respective items further showed a highly similar loading pattern in the EFA with seven factors, we combined them to form the being-moved scale.

Whereas the feeling of sublimity did not load on the awe factor in the EFA with 24 factors, it produced a loading pattern that was highly similar to that of awe in the EFA with seven factors. As these two items were also conceptually closely related [183], we combined the feelings of sublimity and awe to form the awe scale.

Because joy is of great interest within the field of aesthetic emotions and is included in the vast majority of existing measures of aesthetic emotions, we decided to include a separate joy scale. This decision is supported by the findings of [183] that happiness is clearly distinct from humor. Moreover, prior studies have revealed that, while positive emotions are in general less distinct from one another than negative emotions [155], there are important differences between joy and amusement [184].

Finally, as intellectual challenge does not necessarily lead to insight, we considered it important to have a separate insight scale [143, 144]. The study by Hosoya and colleagues [183] further showed that people clearly distinguish between terms designating insight and terms designating intellectual challenge.

Table 4 presents the item statistics and Cronbach’s alphas for the 21 Aesthemos scales. Table 5 shows the correlations of the means of the 21 scales. Given that each scale consists of only two items, the Cronbach’s alpha coefficients are relatively high (between α = .55 for awe and α = .85 for humor).

**Table 4:** Item descriptives and Cronbach’s alphas of the Aesthetic Emotions Scale (Aesthemos).

Item	Subscale	*M*	*SD*	*r*_it_	α
25 Liked it	1 Feeling of beauty/liking	4.05	0.90	.65	.73
28 I found it beautiful	1 Feeling of beauty/liking	3.58	1.09	.65	
19 Was impressed	2 Fascination	3.55	1.11	.67	.77
75 Fascinated me	2 Fascination	3.45	1.16	.67	
15 Felt deeply moved	3 Being moved	3.10	1.17	.68	.77
12 Touched me	3 Being moved	3.66	1.08	.68	
9 I found it sublime	4 Awe	2.27	1.17	.43	.55
51 Felt awe	4 Awe	2.25	1.14	.43	
37 Felt something wonderful	5 Enchantment	2.58	1.17	.72	.79
50 Was enchanted	5 Enchantment	2.35	1.23	.72	
33 Made me feel nostalgic	6 Nostalgia	2.16	1.18	.65	.73
41 Made me feel sentimental	6 Nostalgia	2.48	1.18	.65	
13 Delighted me	7 Joy	3.48	1.09	.78	.84
11 Made me happy	7 Joy	3.08	1.27	.78	
71 Amused me	8 Humor	2.87	1.16	.78	.85
59 Was funny to me	8 Humor	2.65	1.21	.78	
2 Spurred me on	9 Vitality	2.95	1.12	.70	.80
3 Invigorated me	9 Vitality	3.35	1.08	.70	
53 Motivated me to act	10 Energy	2.55	1.11	.53	.66
39 Energized me	10 Energy	2.61	1.16	.53	
7 Calmed me	11 Relaxation	2.30	1.14	.48	.60
74 Relaxed me	11 Relaxation	2.84	1.19	.48	
31 Baffled me	12 Surprise	2.83	1.09	.64	.75
44 Surprised me	12 Surprise	2.99	1.07	.64	
34 Made me curious	13 Interest	3.12	1.16	.57	.69
46 Sparked my interest	13 Interest	3.53	1.01	.57	
17 Challenged me intellectually	14 Intellectual challenge	3.00	1.16	.69	.78
24 Was mentally engaged	14 Intellectual challenge	2.88	1.14	.69	
23 Felt a sudden insight	15 Insight	2.20	1.01	.51	.64
45 Sensed a deeper meaning	15 Insight	2.90	1.20	.51	
63 I found it distasteful	16 Feeling of ugliness	1.34	0.73	.68	.71
58 I found it ugly	16 Feeling of ugliness	1.45	0.82	.68	
64 Felt indifferent	17 Boredom	1.54	0.90	.70	.75
68 Bored me	17 Boredom	1.60	0.94	.70	
61 Felt confused	18 Confusion	1.74	0.99	.68	.75
69 Was unsettling to me	18 Confusion	1.57	0.90	.68	
35 Made me aggressive	19 Anger	1.56	0.96	.70	.75
8 Made me angry	19 Anger	1.64	1.01	.70	
16 Worried me	20 Uneasiness	2.25	1.21	.67	.75
5 Felt oppressive	20 Uneasiness	2.39	1.27	.67	
67 Made me sad	21 Sadness	2.13	1.10	.63	.73
20 Made me feel melancholic	21 Sadness	2.54	1.15	.63	

*Note*. Item discriminations *r*_it_ are polychoric correlations between the items.

The Aesthemos, as described in this article, can be used without charge for academic research purposes by qualified researchers, provided this paper is cited in full in any publication using results obtained with the scale or any adaptation thereof for specific purposes. Any commercial use requires special authorization by the senior author, E-Mail: Klaus.Scherer@unige.ch

**Table 5:** Intercorrelations of Aesthemos subscale means.

Subscale	1	2	3	4	5	6	7	8	9	10	11	12	13	14	15	16	17	18	19	20
1 Feeling of beauty/liking																				
2 Fascination	**.62**																			
3 Being moved	**.52**	**.61**																		
4 Awe	**.33**	**.46**	**.39**																	
5 Enchantment	**.60**	**.58**	**.46**	**.46**																
6 Nostalgia	**.29**	**.26**	**.25**	**.34**	**.43**															
7 Joy	**.67**	**.43**	**.30**	**.26**	**.64**	**.26**														
8 Humor	**.32**	**.16**	.06	-.04	**.28**	**.20**	**.45**													
9 Vitality	**.53**	**.47**	**.37**	**.35**	**.61**	**.22**	**.64**	**.30**												
10 Energy	**.40**	**.44**	**.35**	**.34**	**.57**	**.32**	**.44**	**.17**	**.55**											
11 Relaxation	**.46**	**.29**	.11	**.25**	**.41**	**.32**	**.55**	**.20**	**.31**	**.32**										
12 Surprise	**.37**	**.55**	**.43**	**.30**	**.40**	**.21**	**.26**	**.24**	**.34**	**.31**	.15									
13 Interest	**.49**	**.63**	**.51**	**.32**	**.42**	**.22**	**.28**	**.12**	**.36**	**.40**	**.17**	**.49**								
14 Intellectual challenge	.10	**.35**	**.28**	**.28**	**.16**	**.21**	.00	-.08	.10	**.18**	-.05	**.32**	**.44**							
15 Insight	**.25**	**.48**	**.50**	**.38**	**.38**	**.32**	.15	.06	**.25**	**.38**	.15	**.43**	**.55**	**.52**						
16 Feeling of ugliness	**-.43**	**-.25**	**-.23**	-.05	**-.25**	-.09	**-.34**	-.03	**-.32**	**-.20**	**-.22**	-.04	**-.23**	-.01	-.07					
17 Boredom	**-.50**	**-.45**	**-.46**	-.09	**-.29**	-.07	**-.37**	-.14	**-.38**	**-.28**	**-.15**	**-.25**	**-.39**	-.14	**-.26**	**.52**				
18 Confusion	**-.28**	-.01	.05	.07	**-.16**	.01	**-.34**	-.09	**-.22**	-.08	**-.28**	**.20**	.01	**.25**	.11	**.45**	**.23**			
19 Anger	**-.45**	**-.18**	-.06	-.09	**-.27**	-.10	**-.47**	**-.19**	**-.30**	-.14	**-.36**	.00	-.09	.10	.06	**.45**	**.33**	**.45**		
20 Uneasiness	**-.32**	.02	**.15**	.03	**-.26**	-.05	**-.49**	**-.26**	**-.30**	-.11	**-.36**	.12	.10	**.31**	**.26**	**.30**	.11	**.55**	**.47**	
21 Sadness	.02	**.24**	**.40**	**.17**	.07	**.36**	**-.23**	-.09	-.06	.13	-.10	**.20**	**.23**	**.27**	**.40**	.07	-.08	**.34**	**.28**	**.48**

*Note*. All significant correlations on a Holm-corrected alpha level of *p* < .05 are printed in bold.

## Discussion

Using a combined theoretical and empirical approach, we developed a new questionnaire that can be applied across various domains ranging from the prototypical arts to design, architecture, and nature: the Aesthetic Emotions Scale (Aesthemos). The Aesthemos comprises 21 subscales with two items each (i.e., 42 items in total) to measure specific aesthetic emotions.

Overall, the 21 subscales of the Aesthemos and their seven superordinate factors support the notion that aesthetic experiences are not well represented using only two bipolar dimensions conceived of as valence and arousal. In consequence, it does not make much sense to ask whether the emotions that were experienced were positive or negative, and whether the person felt energized or calm. Rather, aesthetically pleasurable experiences often involve mixtures of positive and negative valence and can be experienced as both arousing and relaxing (see the positive subscale intercorrelations in Table 5). To do justice to the emotionally complex experience underlying aesthetic pleasure, we need to assess a broad range of specific aesthetic emotions and see how they combine in individual aesthetic experiences.

### Aesthemos subscales and subclasses of aesthetic emotions

From 24 a priori emotion categories, we retained 21 categories in the final questionnaire. The 21 Aesthemos subscales were largely supported by an EFA with 24 extracted factors and an EFA with seven factors. We combined the feeling of beauty and liking and dropped the disliking and flow/absorption categories. Because the three items intended to measure flow/absorption did not measure the same construct but rather a mixture of self-forgetfulness, energization, and captivation, we did not include the respective scale and items in the final questionnaire. On the one hand, our findings suggested that absorption or immersion may not have a distinct emotional quality and thus may not be an aesthetic emotion to begin with. On the other hand, short scales measuring transportation [185], absorption [186], and flow [187] are available. Researchers interested in immersion could employ one of these scales together with the Aesthemos.

Our factor analysis pointed to seven factors as a parsimonious representation of aesthetic emotional experience. Nevertheless, for theoretical reasons, we decided to develop a questionnaire that allows for a fine-grained assessment of aesthetic emotions on 21 subscales. First, the analyses were done on a single data set comprising specific events and participants. The different facets may show smaller correlations when other events or participants are considered. Second, although the correlations are often high, the mean scores can be quite different (see Table 4). Researchers interested in the profile of aesthetic emotions that are elicited by specific events would obtain more information based on 21 facets than on seven factor scores. Applied researchers and event managers might be interested in the effect of an event on specific aesthetic emotions.

For the following discussion, we have sorted the 21 Aesthemos subscales into the broad subclasses of aesthetic emotions identified in the introduction. Our notion that these subclasses indeed capture major facets of aesthetic evaluation also received empirical support through our EFA with seven factors. While our theoretical categorization of individual aesthetic emotions is not completely in line with the factor loading pattern, the results still show that prototypical aesthetic emotions are distinct from pleasing emotions, epistemic emotions, and negative emotions.

#### Prototypical aesthetic emotions

The Aesthemos offers several subscales that represent the spectrum of emotions that have been considered as prototypical aesthetic emotions [5, 7, 24, 28, 29, 114]. These include (1) feeling of beauty/liking, (2) fascination, (3) being moved, and (4) awe. These emotions capture aesthetic appreciation irrespective of the pleasingness (in terms of purely positive affective valence) of the aesthetic experience.

Although we had a priori included the feeling of beauty and liking as two separate categories, our findings showed that they were inseparable in this data set, which is why we combined them into one scale. This is in line with other studies that have found that beauty and liking are highly correlated [188, 189]. Nevertheless, it should be noted that beauty is by no means a prerequisite for liking [190, 191], that is, stimuli can be liked although they are not beautiful. The close connection between beauty and liking in our data may also be related to our use of the German term *schön*, which means beautiful but is also used to indicate feelings of liking or pleasure. Future research is therefore needed to further explore the differences between the feeling of beauty and liking and the possibility of eliciting verbal responses that separate these constructs.

The items we had formulated to measure captivation did not form a single coherent scale. Therefore, we focused instead on fascination, an emotion that is central to theoretical accounts of aesthetic experience [24, 29, 30]. While we had originally included *fascinated* as a potential item to assess interest, our findings showed that fascination is more closely linked to being impressed and overwhelmed and is thus part of the prototypical aesthetic emotions.

Based on our literature review, we had also considered subscales (5) enchantment and (6) nostalgia as part of the prototypical aesthetic emotions. However, the seven-factor EFA did not provide clear support for this decision. While the feeling of beauty/liking, fascination, being moved, and awe loaded on one factor (F7_2), enchantment and nostalgia loaded with other subscales (to be discussed further below).

#### Pleasing emotions

Our theoretical subclass of pleasing emotions included all emotions with positive affective valence. Such emotions are assessed by the Aesthemos subscales (7) joy, (8) humor, (9) vitality, (10) energy, and (11) relaxation.

The joy and humor subscales help to study aesthetic experiences that are perceived as fun and thus most clearly represent the pleasingness of aesthetic experience. In line with this conclusion, Weisfeld [192] suggested, in a discussion of humor as an aesthetic emotion, that the special appeal of humor lies in its unalloyed pleasure. Even when the butt of a joke suffers, people may not feel sympathy or pity, but rather amusement.

A connection between joy and humor was also supported by our seven-factor EFA showing that both scales load on an *amusement* factor (F7_7). This analysis also suggested that the subclass of pleasing emotions needed to be more finely differentiated. Amusement, animation, and relaxation (together with nostalgia) formed three separate factors.

However, the *animation* (F7_4) and *nostalgia/relaxation* (F7_5) factors are not as readily interpretable as the other five factors. The common features of the subscales included are high (F7_4) and low (F7_5) activation. Yet each factor contains one scale (enchantment and nostalgia, respectively) that cannot be reduced to activation potential.

It is possible to explain the inclusion of enchantment together with energy and vitality in F7_4 in terms of *animation*. That is, energy, vitality, and enchantment all imply the feeling of spirit or energy, yet the energy source is different. Our energy subscale reflects being energized toward the attainment of an objective: The person is energized to perform some action. The energy source, thus, is the motivational pull from an activity or future prospect.

In contrast, vitality has been characterized as physical and mental energy available to the self or the feeling of aliveness [152, 193]. Vitality emanates from the self and can be used for self-regulation. In line with this suggestion, Ryan and Frederick [193] showed that items indicative of vitality are separable from items related to having purpose and direction.

When people feel enchanted, the source of energy is some magical or spiritual power. Enchantment not only emerged as an emotion potentially experienced in response to art (see Table 1), but it is also increasingly recognized as an important facet of the consumer experience. For instance, studies have revealed spectacular, immersive, and ritualized dimensions underlying the out-of-the-ordinary experiences of museoparks [194], ballparks [195], and malls [196]. In contrast to energy and vitality, the enchantment items showed cross-loadings on the *prototypical aesthetic emotions* factor (F7_2). This suggests that enchantment may well form a factor together with the other prototypical aesthetic emotions in future studies rather than being combined with energy and vitality.

The inclusion of the relaxation and nostalgia subscales in one factor may be due to the potential these emotions have to restore an individual’s peace of mind. Both relaxation and nostalgia can help cope with life’s minor and major hassles, ranging from daily stress to major turning points in life, albeit in different ways. While relaxation (see [21, 149–151]) has more immediate affect-regulatory functions and restores the capacity for directed attention, nostalgia emerged as a resource that helps maintain self-continuity [197] and strengthen a sense of meaning in life [198].

Such positive aspects of nostalgia may also explain its prominence in marketing research (for an overview of studies on nostalgia and consumer behavior, see [199]), at least since the seminal articles of Belk [200], Havlena and Holak [201], and Holbrook and Schindler [202]. Researchers in this area have accorded nostalgic brands an important role in the self-regulation of mood, and, more specifically, mood repair [203]. Beyond immediate affect-regulatory functions, nostalgic products have been recognized as means to develop, sustain, and recreate consumers’ identities [199].

Nevertheless, nostalgia is a mixed emotion that can have negative consequences when people do not have sufficient control over their nostalgia or longing [130, 131]. In our study, nostalgia items tended to cross-load on the *sadness* factor (F7_6; but note that these loadings were not significant at *p* < .05), which underscores this mixed emotional nature rather than a purely relaxing nature of nostalgia. Moreover, the emergence of a factor including relaxation and nostalgia may be limited to the context of aesthetic emotions. Individuals may experience greater control over feelings of nostalgia and longing in response to the arts and related domains. Therefore, the adaptive aspects of nostalgia may predominate in such contexts, which contributes to similar ratings of relaxation and nostalgia as aesthetic emotions with restorative and coping functions.

In light of these considerations, the animation and nostalgia/relaxation factors may not be consistently replicated in future research (but we note that nostalgia and peacefulness also loaded on a common superordinate factor in the GEMS [70]). This further underscores the necessity to study aesthetic experience on the level of the 21 subscales rather than trying to limit this rich information to subclasses of aesthetic emotions whose boundaries are not clearly demarcated.

#### Epistemic emotions

The Aesthemos includes the following scales to measure specific epistemic emotions [5, 10, 132, 154] and pleasures of the mind [118]: (12) surprise, (13) interest, (14) intellectual challenge, and (15) insight. In line with the distinction between pleasingness and meaning as sources of enjoyment [2, 110, 139, 140], we identified an epistemic emotions factor (F7_3) representing the search for and finding of meaning during aesthetic experiences. Items of the intellectual challenge, interest, and insight subscales loaded on this factor. In contrast, items of the surprise subscale had cross-loadings on the epistemic emotions factor, but primarily loaded together with the prototypical aesthetic emotions (F7_2). This illustrates an important difference between surprise and the other epistemic emotions: surprise is a short-lived response to something that is novel and unexpected, but does not depend on a positive evaluation of one’s potential to cope with the schema incongruity that led to the surprise [132, 156]. In contrast, intellectual challenge, interest, and insight depend on one’s (potential) ability to understand and thus satisfy the drive for sense-making [31, 119, 120]. This also explains why items assessing confusion, another knowledge emotion based on Silvia’s classification [132], loaded on the negative emotions factor rather than together with the other epistemic emotions. If a person evaluates her or his potential to understand a surprising event as insufficient, surprise turns into confusion. This ambivalent nature of surprise may explain its loading on the prototypical aesthetic emotions factor together with other mixed and potentially ambivalent emotions. Like fascination and awe, surprise captures attention and orients people to its elicitor, regardless of whether the elicitor is potentially pleasing or comprehensible.

#### Negative emotions

In line with our conclusion that a measure of aesthetic emotions needs to include negative emotions, the Aesthemos subscales encompass a broad range of these emotions: (16) feeling of ugliness, (17) boredom, (18) confusion, (19) anger, (20) uneasiness, and (21) sadness. In contrast to the remaining Aesthemos subscales, these emotions often are felt during aesthetic experiences that not only are unpleasant but also contribute to a negative evaluation regarding aesthetic merit. In line with this suggestion, some studies have found negative associations of boredom, distress, fear, or anger with liking and aesthetic appeal ratings [50, 105, 204, 205]. The respective subscales are thus important for studying negative reactions to the perceived aesthetic virtues of stimuli, such as those highlighted by Silvia [132].

Nevertheless, it is important to note that the negative emotion items formed a separate factor (F7_1) rather than loading negatively on the *prototypical aesthetic emotions* factor (F7_2). Together with the emergence of a separate *sadness* factor (F7_6), this shows that negative aesthetic emotions do not merely indicate displeasure.

There are two possible explanations of why the sadness subscale represents a factor of its own rather than loading on the *negative emotions* factor. On the one hand, this may be due to the inclusion of melancholy in the sadness scale. Melancholy can be considered as an aesthetic emotion that is different from sadness and depression [117]. In contrast to the latter, melancholy is a complex emotion that includes the pleasure of indulgent reflection in addition to feelings of sadness, loneliness, and emptiness. Thus, the sadness subscale may measure sadness as a genuine aesthetic emotion.

On the other hand, the finding of a separate *sadness* factor may point to a special role of sadness (even without the inclusion of melancholy), in contrast to anger and fear, as an aesthetically pleasurable emotion. Goldstein [206] has suggested that sadness experienced in response to movies rather than in real life is unadulterated by anxiety and therefore enjoyable. Indeed, studies have shown that sadness contributes to the aesthetic appeal of music, whereas fear and anger evoked by music are negatively related to liking and beauty ratings [204, 205].

When interpreting the *sadness* factor, we also need to consider the cross-loadings of items indicative of being moved on this factor. Whereas sadness and being moved emerged as separate components in both EFAs, being moved showed almost equal loadings on the *prototypical aesthetic emotions* factor (F7_2) and the *sadness* factor (F7_6). In contrast, the sadness items had no cross-loadings on F7_2. This once more suggests that the associated feeling of being moved explains the pleasurable nature of sadness during aesthetic experience [63]. In contrast, anger and uneasiness are not typically associated with being moved [123].

Nevertheless, even clearly unpleasant emotions may contribute to subsequent aesthetic pleasure (cf. [64]). There is some evidence for a role of negative affect in general and sadness or fear more specifically as triggers of mixed aesthetic emotions. When they were in a negative mood, people experienced greater nostalgia in general [207] and in response to music [208]. Nostalgia proneness further related positively to dispositional sadness and neuroticism [208]. Induced fear produced increased feelings of sublimity in response to visual artworks [209]. Moreover, people were more touched by a tragic story after mortality-salience induction (thinking about their own death [210]), which also likely elicited some fear [211]. Feeling negative emotions during aesthetic experience may thus contribute to the subsequent emergence and enhancement of mixed emotions. As the perception of aesthetically relevant stimuli unfolds across time, it is important to measure the occurrence of purely negative emotions to capture the full impact and appeal of such stimuli. Depending on the nature and sequence of emotions during aesthetic experience, negative emotions may contribute to aesthetic displeasure or aesthetic pleasure.

### Limitations

Some limitations of this research and the resulting Aesthemos must be acknowledged. First, we assessed our participants’ aesthetic emotions only after the event rather than through an on-line assessment of emotions during the event. Considering the length of the emotion questionnaire (75 items), it would not have been feasible to collect repeated assessments. Nevertheless, this procedure may have led to a picture of aesthetic emotions that is reduced in terms of variety. For instance, our findings reflect that aesthetic appreciation, in retrospect, resulted from a well-orchestrated range of various positive, mixed, and possibly also negative emotions. When assessed from moment to moment, correlations between different emotions will likely become smaller.

A second and related problem is that the emotional experience of a stimulus changes across time. Our subsequent assessment did not allow us to determine which specific scene or moment produced the emotions that were reported. Moreover, in the case of museum exhibitions, we cannot determine which object or artwork on display gave rise to an emotion.

Third, the Aesthemos relies on self-reports. Responses may thus be influenced by genre expectations, the emotions expressed in an artwork, social desirability, or response tendencies in addition to actually felt emotions. Moreover, where scientific definitions of emotions differ from the vernacular (as is the case for the feeling of beauty and liking discussed above, but probably also for fascination and the feeling of the sublime), data collected with the Aesthemos cannot be expected to match predictions from theoretical aesthetics. It will thus be necessary to use additional methods to test such predictions. For instance, researchers could experimentally manipulate stimuli based on theoretical predictions of how to make them more or less beautiful or sublime and then study the effect of such manipulation on the emotion profile reported in the Aesthemos. In addition, self-report data could be combined with physiological and/or behavioral measures (as discussed below as one direction for future research).

Fourth, the broad scope of emotions and domains covered by the Aesthemos can be considered a weakness. Our new tool samples a diverse set of emotions to also include emotions that are not highly similar to the prototypical aesthetic emotions. At the same time, we did not include emotions that are relevant only within some aesthetic domains and irrelevant in others. This leads to two potential drawbacks. On the one hand, the Aesthemos may not include emotions that are central for capturing affective reactions to stimuli within one specific domain or genre. For instance, it does not include a nuanced measure of emotional responses to primarily cute, disgusting, or horrific stimuli. Past studies have highlighted the relevance of love and feelings of warmth in marketing and consumer research [19, 96, 106, 212]. Tender feelings and a characterization as “romantic” are, further, characteristic of music and poems (see [175] on the similarity of terms used to designate the aesthetic appeal of music and poems). Moreover, visual stimuli that appeal to children and many adults as well often draw upon emotions elicited by cuteness. Cuteness is linked to vulnerability and is thus in part captured by the being moved subscale [213]. Cuteness is also related to liking/attraction. Nevertheless, the Aesthemos does not include emotion terms that indicate feelings of tenderness.

For studies of narrative formats like literature or film, additional items to measure feelings associated with suspense may be added. For instance, Knoop and colleagues [175] found that participants used the term *suspenseful* more frequently than *beautiful* to designate the aesthetic appeal of novels. Researchers interested in the specific genre of horror films may want to add emotions like the thrill or “kick” experienced during such movies [84]. While the fascination, interest, surprise, and uneasiness subscales of the Aesthemos tap into feelings associated with suspense or an adrenalin kick, these latter constructs could be measured more explicitly in future studies.

Turning to marketing and advertising, it is important to note that the Aesthemos focuses on emotional dimensions of aesthetically appreciating inherent qualities of consumer products or services rather than on the pragmatic uses and benefits of such products and services. As revealed by the respective measures in Table 1, additional emotions need to be considered when predicting actual consumer behavior.

Thus, the Aesthemos may have to be extended in these specific contexts to include subscales for tenderness, suspension, or thrill. Alternatively, when researchers are interested in only one domain, they can select from among the aesthetic emotion measures presented in Table 1 those that are more closely matched to the specific nature of the stimuli in that domain.

On the other hand, the Aesthemos may be perceived as including too many emotions that are of peripheral importance to aesthetic evaluation within a specific domain, which may lead to lower face validity for the instrument. For instance, studies of experiences of nature have included emotions like vitality [152], relaxation [21, 150], and awe [95, 214]. In contrast, emotions like humor or anger seem to have little relevance for the aesthetic experience of nature.

### Directions for future research

Despite having some limitations, the Aesthemos opens up new possibilities for future research. It provides researchers with a highly nuanced emotion profile that allows for a more detailed investigation of the influences of multiple factors relevant to aesthetic perception and judgment, such as features of the stimulus, recipient, and situation [3, 5, 215].

For instance, the Aesthemos can be employed to compare emotion profiles across different domains, such as music, painting, literature, nature experiences, and so forth. While prior research has revealed differences in the aesthetic emotions central to different domains [216], the research had to rely on tools that were developed for a specific domain (in this case music) and may have missed aesthetic emotions that are important in other domains. Future research using the Aesthemos in different domains could offer guidelines for the selection of subscales to be included when studying a specific domain and thus allow reducing the number of emotions of little relevance being assessed in a study.

To further test and validate the Aesthemos, it should be combined with other measures indicative of aesthetic emotions beyond self-report questionnaires. These include behavioral observations in field studies or in the lab (e.g., eye movements, time spent in a specific location, viewing or reading time, preferences for specific environments, movement speed and pattern) and physiological measures (e.g., facial muscle activity, pupillary dilation, patterns of brain activation, chills/goosebumps, skin conductance, heart rate, respiration rate, or body temperature). Prior studies have revealed connections of self-reported aesthetic emotions with behavioral and physiological measures (see, e.g., [217] for music and film, [218] for visual art, [92] for museum exhibitions, and [219] for poetry). The Aesthemos can help increase our knowledge of whether specific aesthetic emotions are more closely linked to a specific objective measure than others. For instance, while aesthetic chills have been considered to be global indicators of peak emotional responses (e.g., [220–223]), other studies have suggested that chills are particularly related to specific emotions like being moved, awe, and the epistemic emotions [28, 67, 120, 224, 225]. The Aesthemos could be employed to test whether these latter emotions are more predictive of chills than are other emotions.

The Aesthemos can also help further our understanding of differences between the aesthetic experiences of experts and laypersons [13, 226–228]. While attenuated emotional responses to positive and negative artworks have been found in experts [48], this finding may hold true only for the negative and pleasing emotions studied. It is possible that experts show more intense prototypical aesthetic emotions and epistemic emotions (e.g., Silvia [13] found greater interest among experts compared to novices), which the Aesthemos covers in their full spectrum.

Another possibility for future research would be to determine which personal characteristics contribute to the experience of specific aesthetic emotions. For instance, empathy [68, 229] and openness to experience [95] facilitate the experience of aesthetic emotions like being moved, awe, sublimity (including transcendence, nostalgia, and peacefulness), and interest. The Aesthemos could help determine which personality traits are related to which aesthetic emotions and whether these personality-emotion links account for interindividual differences in genre or domain preferences.

Moreover, our new assessment tool could be used to increase our understanding of the effects of mood and emotional states on subsequent aesthetic perceptions and evaluations. Research could build on past findings [208, 209, 230] and test which aesthetic emotions are enhanced and which are diminished in people who feel sad, angry, fearful, or happy.

Finally, future research could determine how well suited the Aesthemos is to assess emotions in response to odors, tastes, or, more generally, stimuli that appeal to both distance and contact senses. Even though the resulting emotions are not prototypically aesthetic, it is informative for consumer research to assess these emotions. Extant studies of emotional responses to odors [46, 231] and food [47, 232] include emotions like relaxation, joy, energy, vitality, surprise, interest, nostalgia, boredom, sadness, uneasiness, and anger. As is evident from this list, there is considerable overlap with the Aesthemos subscales. It may thus be fruitful to consider our new measure in these contexts as well, to gain a clearer understanding of the spectrum of consumption-related and more narrowly defined aesthetic emotions.

### Conclusion

We have introduced the Aesthemos (as presented in S1 Appendix) as a new tool for assessing aesthetic emotions that is applicable across a wide range of domains. It contains 21 subscales that allow for a fine-grained differentiation of the spectrum of emotions most relevant to experiences of the perceived aesthetic appeal of stimuli. In addition to prototypical aesthetic emotions (e.g., the feeling of beauty, fascination, or being moved) and negative emotions (e.g., the feeling of ugliness, boredom, or confusion), our new tool assesses emotions linked to pleasing and sense-making ways of enjoying aesthetics. These include humor, joy, vitality, and relaxation as well as intellectual challenge, interest, and insight. The Aesthemos thus represents the first integrative measure to capture the full range of emotions that can account for the pleasure and displeasure experienced in contexts of aesthetic perception and evaluation/appreciation.

## Supporting information

S1 Appendix The Aesthetic Emotions Scale (Aesthemos). Presents an example of the **Aesthemos** for use in future studies.
(DOCX)

S1 Fig Eigenvalues of the items’ polychoric correlation matrix for all 75 items and the factor analytic model. (DOCX)

S1 Table Initial set of 75 emotion items included in the study and a priori categories. The original items included in the study in German and English along with our a priori categorization into 24 emotion categories.
(DOCX)

S2 Table Events and locations of the field study data collection. (DOCX)

S3 Table Factor structure matrix of an exploratory factor analysis with 24 factors. (DOCX)

S4 Table Factor correlation matrix based on an EFA with 24 factors and oblimin rotation. (DOCX)

S5 Table Factor structure matrix of an exploratory factor analysis with seven factors. (DOCX)

S6 Table Factor correlation matrix based on an EFA with seven factors and oblimin rotation. (DOCX)
