# Supplementary material for: Measuring aesthetic emotions: A review of the literature and a new assessment tool
Source: PLoS One. 2017 Jun 5;12(6):e0178899. doi: 10.1371/journal.pone.0178899 (PMC5459466; doi:10.1371/journal.pone.0178899)
Supplement: S1 Appendix — Presents an example of the Aesthemos for use in future studies. (DOCX) [file pone.0178899.s001.docx]

**S1 Appendix**

**The Aesthetic Emotions Scale (Aesthemos)**

The Aesthemos can be used to assess either the intensity of aesthetic emotions (e.g., for studying momentary aesthetic experience or the experience of a specific stimulus, such as a picture, poem, piece of music, or film scene) or the frequency of experiencing aesthetic emotions during a more prolonged aesthetic experience (e.g., for studying an event as a whole, such as an entire art exhibition, theater performance, or a walk through nature). An example of the Aesthemos assessing intensity is shown below.

*For the frequency version, the following modifications need to be made.*

*Rating instruction:* How often did you feel this emotion?

*Rating scale:* From 1 *never* to 5 *very often*

***Instruction:***

Which emotional effect did ____________ have on you?

For each emotion listed below, please mark the response category that best matches your personal experience. Please only indicate how **you** actually felt. Do not characterize the emotions expressed in ____________ if you did not feel them yourself.

|  | **How intensely did you feel this emotion?** | | | | |
| --- | --- | --- | --- | --- | --- |
| **Emotional feeling** | **1**  **not at all** | **2** | **3** | **4** | **5**  **very** |
| 1 I found it beautiful | □_1_ | □_2_ | □_3_ | □_4_ | □_5_ |
| 2 Challenged me intellectually | □_1_ | □_2_ | □_3_ | □_4_ | □_5_ |
| 3 Delighted me | □_1_ | □_2_ | □_3_ | □_4_ | □_5_ |
| 4 Calmed me | □_1_ | □_2_ | □_3_ | □_4_ | □_5_ |
| 5 Made me curious | □_1_ | □_2_ | □_3_ | □_4_ | □_5_ |
| 6 Liked it | □_1_ | □_2_ | □_3_ | □_4_ | □_5_ |
| 7 Fascinated me | □_1_ | □_2_ | □_3_ | □_4_ | □_5_ |
| 8 Felt something wonderful | □_1_ | □_2_ | □_3_ | □_4_ | □_5_ |
| 9 Invigorated me | □_1_ | □_2_ | □_3_ | □_4_ | □_5_ |
| 10 Was mentally engaged | □_1_ | □_2_ | □_3_ | □_4_ | □_5_ |
| 11 Baffled me | □_1_ | □_2_ | □_3_ | □_4_ | □_5_ |
| 12 I found it ugly | □_1_ | □_2_ | □_3_ | □_4_ | □_5_ |
| 13 Sensed a deeper meaning | □_1_ | □_2_ | □_3_ | □_4_ | □_5_ |
|  | **How intensely did you feel this emotion?** | | | | |
| **Emotional feeling** | **1**  **not at all** | **2** | **3** | **4** | **5**  **very** |
| 14 Felt deeply moved | □_1_ | □_2_ | □_3_ | □_4_ | □_5_ |
| 15 Made me feel melancholic | □_1_ | □_2_ | □_3_ | □_4_ | □_5_ |
| 16 Energized me | □_1_ | □_2_ | □_3_ | □_4_ | □_5_ |
| 17 Made me angry | □_1_ | □_2_ | □_3_ | □_4_ | □_5_ |
| 18 Was enchanted | □_1_ | □_2_ | □_3_ | □_4_ | □_5_ |
| 19 Bored me | □_1_ | □_2_ | □_3_ | □_4_ | □_5_ |
| 20 Relaxed me | □_1_ | □_2_ | □_3_ | □_4_ | □_5_ |
| 21 Felt a sudden insight | □_1_ | □_2_ | □_3_ | □_4_ | □_5_ |
| 22 Amused me | □_1_ | □_2_ | □_3_ | □_4_ | □_5_ |
| 23 Made me sad | □_1_ | □_2_ | □_3_ | □_4_ | □_5_ |
| 24 Felt confused | □_1_ | □_2_ | □_3_ | □_4_ | □_5_ |
| 25 Made me aggressive | □_1_ | □_2_ | □_3_ | □_4_ | □_5_ |
| 26 Made me feel sentimental | □_1_ | □_2_ | □_3_ | □_4_ | □_5_ |
| 27 Worried me | □_1_ | □_2_ | □_3_ | □_4_ | □_5_ |
| 28 Made me feel nostalgic | □_1_ | □_2_ | □_3_ | □_4_ | □_5_ |
| 29 Surprised me | □_1_ | □_2_ | □_3_ | □_4_ | □_5_ |
| 30 Felt oppressive | □_1_ | □_2_ | □_3_ | □_4_ | □_5_ |
| 31 I found it sublime | □_1_ | □_2_ | □_3_ | □_4_ | □_5_ |
| 32 Spurred me on | □_1_ | □_2_ | □_3_ | □_4_ | □_5_ |
| 33 Felt indifferent | □_1_ | □_2_ | □_3_ | □_4_ | □_5_ |
| 34 Was impressed | □_1_ | □_2_ | □_3_ | □_4_ | □_5_ |
| 35 I found it distasteful | □_1_ | □_2_ | □_3_ | □_4_ | □_5_ |
| 36 Touched me | □_1_ | □_2_ | □_3_ | □_4_ | □_5_ |
| 37 Was unsettling to me | □_1_ | □_2_ | □_3_ | □_4_ | □_5_ |
| 38 Sparked my interest | □_1_ | □_2_ | □_3_ | □_4_ | □_5_ |
| 39 Made me happy | □_1_ | □_2_ | □_3_ | □_4_ | □_5_ |
| 40 Felt awe | □_1_ | □_2_ | □_3_ | □_4_ | □_5_ |
| 41 Motivated me to act | □_1_ | □_2_ | □_3_ | □_4_ | □_5_ |
| 42 Was funny to me | □_1_ | □_2_ | □_3_ | □_4_ | □_5_ |
