# Supplementary material for: Measuring aesthetic emotions: A review of the literature and a new assessment tool
Source: PLoS One. 2017 Jun 5;12(6):e0178899. doi: 10.1371/journal.pone.0178899 (PMC5459466; doi:10.1371/journal.pone.0178899)
Supplement: S1 Fig — (DOCX) [file pone.0178899.s002.docx]

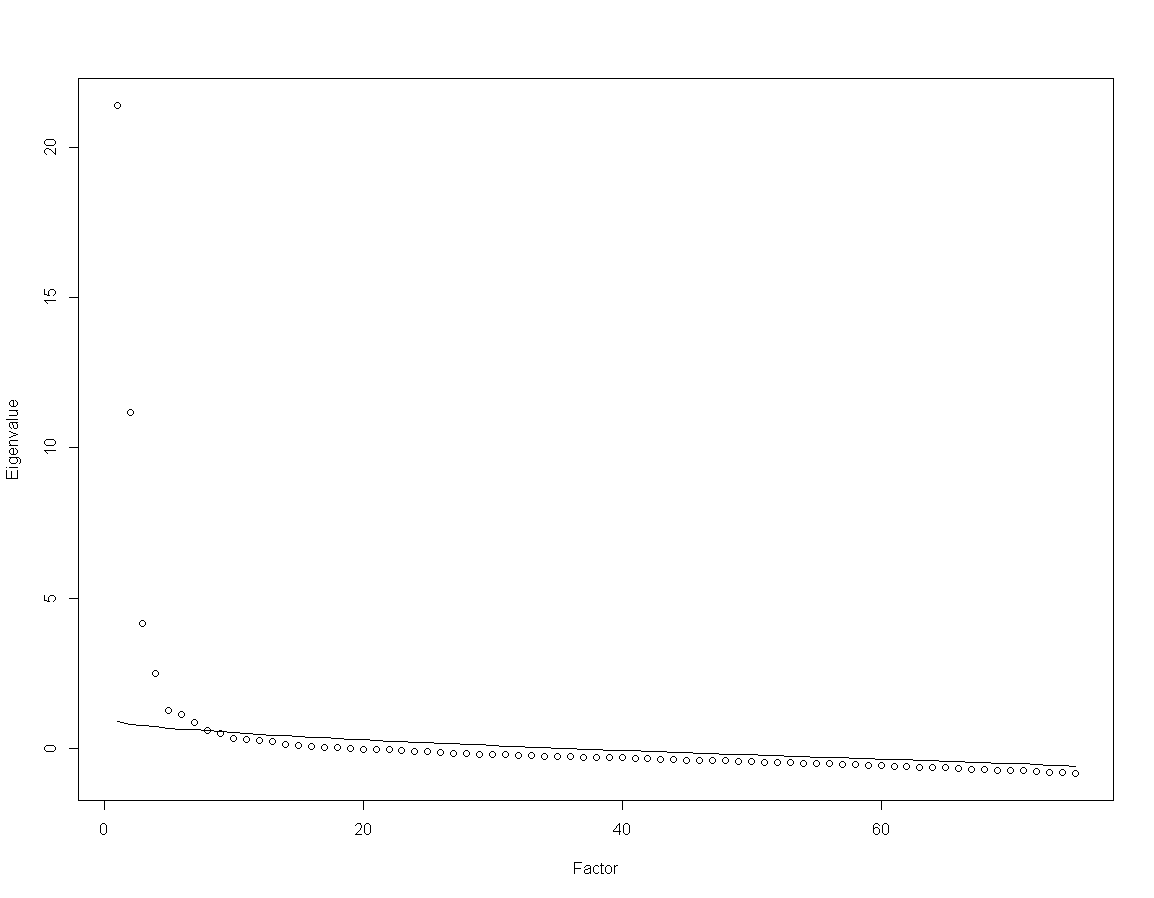


**S1 Figure. Eigenvalues of the Items’ Polychoric Correlation Matrix for All 75 Items and the Factor Analytic Model.**

*Note.* The curved line indicates the expected eigenvalues based on a parallel analysis.
