# Supplementary material for: Measuring aesthetic emotions: A review of the literature and a new assessment tool
Source: PLoS One. 2017 Jun 5;12(6):e0178899. doi: 10.1371/journal.pone.0178899 (PMC5459466; doi:10.1371/journal.pone.0178899)
Supplement: S1 Table — The original items included in the study in German and English along with our a priori categorization into 24 emotion categories. (DOCX) [file pone.0178899.s003.docx]

**S1 Table. Initial Set of 75 Emotion Items Included in the Study and A Priori Categories.**

| **#** | **Original German Item** | **English Translation** | **A Priori Category** |
| --- | --- | --- | --- |
| ***Prototypical aesthetic emotions*** | | | |
| **28** | **Empfand ich als schön** | **I found it beautiful** | **Feeling of Beauty** |
| 48 | Empfand ich als anmutig | I found it graceful | Feeling of Beauty |
| 72 | Empfand ich als harmonisch | I found it harmonious | Feeling of Beauty |
| 14 | Empfand ich als vollkommen | I found it perfect | Feeling of Beauty |
| **25** | **Gefiel mir** | **Liked it** | **Liking/Attraction** |
| 29 | Zog mich an | Was attracted | Liking/Attraction |
| 30 | Begeisterte mich | Made me feel enthusiastic | Liking/Attraction |
| 55 | Empfand ich als angenehm | I found it pleasant | Liking/Attraction |
| 65 | War hingerissen | Was enraptured | Liking/Attraction |
| 6 | Packte mich | Gripped me | Captivation |
| **19** | **War beeindruckt** | **Was impressed** | **Captivation** |
| 49 | War überwältigt | Was overwhelmed | Captivation |
| **12** | **Berührte mich** | **Touched me** | **Being Moved** |
| **15** | **War ergriffen** | **Felt deeply moved** | **Being Moved** |
| 66 | Bewegte mich | Moved me | Being Moved |
| **9** | **Empfand ich als erhaben** | **I found it sublime** | **Awe** |
| 32 | Empfand Demut | Felt humbled | Awe |
| **51** | **Empfand Ehrfurcht** | **Felt awe** | **Awe** |
| **37** | **Fühlte etwas Wunderbares** | **Felt something wonderful** | **Enchantment/Wonder** |
| **50** | **War wie verzaubert** | **Was enchanted** | **Enchantment/Wonder** |
| 70 | Versetzte mich in träumerische Stimmung | Put me in a dreamy mood | Enchantment/Wonder |
| 1 | Weckte in mir Sehnsucht | Filled me with longing | Nostalgia/Longing |
| **33** | **Machte mich nostalgisch** | **Made me feel nostalgic** | **Nostalgia/Longing** |
| **41** | **Weckte in mir sentimentale Gefühle** | **Made me feel sentimental** | **Nostalgia/Longing** |
| ***Pleasing emotions*** | | | |
| 4 | Stimmte mich fröhlich | Made me cheerful | Joy |
| **11** | **Machte mich glücklich** | **Made me happy** | **Joy** |
| **13** | **Erfreute mich** | **Delighted me** | **Joy** |
| 43 | Erheiterte mich | Made me merry | Humor |
| **59** | **Belustigte mich** | **Was funny to me** | **Humor** |
| **71** | **Amüsierte mich** | **Amused me** | **Humor** |
| **3** | **Belebte mich** | **Invigorated me** | **Vitality/Arousal** |
| 47 | Wühlte mich auf | Agitated me | Vitality/Arousal |
| 52 | Machte mich munter | Perked me up | Vitality/Arousal |
| **2** | **Beflügelte mich** | **Spurred me on** | **Energy** |
| **39** | **Energetisierte mich** | **Energized me** | **Energy** |
| **53** | **Weckte meinen Tatendrang** | **Motivated me to act** | **Energy** |
| **7** | **Beruhigte mich** | **Calmed me** | **Relaxation** |
| 10 | Machte mich zufrieden | Made me feel content | Relaxation |
| **74** | **Entspannte mich** | **Relaxed me** | **Relaxation** |

| **#** | **Original German Item** | **English Translation** | **A Priori Category** |
| --- | --- | --- | --- |
| ***Epistemic emotions*** | | | |
| **31** | **Verblüffte mich** | **Baffled me** | **Surprise** |
| **44** | **Überraschte mich** | **Surprised me** | **Surprise** |
| 57 | Versetzte mich in Staunen | Astonished me | Surprise |
| **34** | **Machte mich neugierig** | **Made me curious** | **Interest** |
| **46** | **Weckte mein Interesse** | **Sparked my interest** | **Interest** |
| **75** | **Faszinierte mich** | **Fascinated me** | **Interest** |
| **17** | **Forderte mich intellektuell heraus** | **Challenged me intellectually** | **Intellectual Challenge** |
| **24** | **War geistig gefordert** | **Was mentally engaged** | **Intellectual Challenge** |
| 73 | Regte meine Gedanken an | Stimulated my thoughts | Intellectual Challenge |
| **23** | **Fühlte eine plötzliche Einsicht** | **Felt a sudden insight** | **Insight** |
| 42 | Inspirierte mich | Inspired me | Insight |
| **45** | **Spürte einen tieferen Sinn** | **Sensed a deeper meaning** | **Insight** |
| ***Negative emotions*** | | | |
| 27 | Stieß mich ab | Repelled me | Feeling of Ugliness |
| **58** | **Empfand ich als hässlich** | **I found it ugly** | **Feeling of Ugliness** |
| **63** | **Empfand ich als geschmacklos** | **I found it distasteful** | **Feeling of Ugliness** |
| 21 | Behagte mir nicht | Made me feel uncomfortable | Disliking/Displeasure |
| 36 | Missfiel mir | Disliked it | Disliking/Displeasure |
| 60 | Empfand ich als unangenehm | I found it unpleasant | Disliking/Displeasure |
| 38 | Ermüdete mich | Tired me | Boredom |
| **64** | **War mir gleichgültig** | **Felt indifferent** | **Boredom** |
| **68** | **Langweilte mich** | **Bored me** | **Boredom** |
| 26 | Schockierte mich | Was shocking to me | Confusion |
| **61** | **Fühlte mich verwirrt** | **Felt confused** | **Confusion** |
| **69** | **Verstörte mich** | **Was unsettling to me** | **Confusion** |
| **8** | **Stimmte mich ärgerlich** | **Made me angry** | **Anger** |
| **35** | **Machte mich aggressiv** | **Made me aggressive** | **Anger** |
| 40 | Irritierte mich | Irritated me | Anger |
| **5** | **Wirkte auf mich beklemmend** | **Felt oppressive** | **Uneasiness/Fear** |
| **16** | **Beunruhigte mich** | **Worried me** | **Uneasiness/Fear** |
| 56 | Beängstigte mich | Scared me | Uneasiness/Fear |
| **20** | **Stimmte mich melancholisch** | **Made me feel melancholic** | **Sadness** |
| 22 | Fühlte mich bedrückt | Felt depressed | Sadness |
| **67** | **Stimmte mich traurig** | **Made me sad** | **Sadness** |
| ***Self-forgetful emotions*** | | | |
| 18 | War mir meiner selbst nicht bewusst | Was not aware of myself | Flow/Absorption |
| 54 | Fühlte mich in der Erfahrung aufgehen | Felt absorbed in the experience | Flow/Absorption |
| 62 | Spürte die Zeit wie im Flug vergehen | Felt that time was flying | Flow/Absorption |

*Note*. Numbers in front of the items indicate the item order in the questionnaire. The 42 items included in the final AESTHEMOS are highlighted in bold.
