# Supplementary material for: Measuring aesthetic emotions: A review of the literature and a new assessment tool
Source: PLoS One. 2017 Jun 5;12(6):e0178899. doi: 10.1371/journal.pone.0178899 (PMC5459466; doi:10.1371/journal.pone.0178899)
Supplement: S2 Table — (DOCX) [file pone.0178899.s004.docx]

**S2 Table. Events and Locations of the Field Study Data Collection.**

| ***n*** | **Event Type** | **Event** | **Berlin Location** |
| --- | --- | --- | --- |
| 6 | Acrobatics show | FLiP | Chamäleon Theater |
| 14 | Concert (pop, jazz, gospel etc.) | A cappella ensemble VocaYou | Café Fincan |
| 38 | Classical concert | Collegium Musicum Summer Concert | Berlin Philharmonic Hall |
| 14 | Organ concert | Rache in c-Moll! [Revenge in C minor!] | Berlin Cathedral |
| 17 | Classical concert | artenius trio Berlin | Orangery of Glienicke Palace |
| 19 | Filmopera | Le trois souhaits ou Les vicissitudes de la vie [The three wishes or the vicissitudes of life] | UNI.T-Theatre of the UdK Berlin |
| 12 | Musical | Hinterm Horizont [Behind the horizon] | Stage Theater at Potsdamer Platz |
| 20 | Swing concert and dance | Harald’s Swing Jam Session | Sally Bowles |
| 28 | Dance theatre | 20 Dancers for the XX Century | Soviet War Memorial in the Treptower Park |
| 20 | Dance theatre | Sun | Haus der Berliner Festspiele, Main Stage |
| 30 | Theatre | Migronauten | TAK Theater im Aufbau Haus |
| 30 | Theatre | The Black Rider | Schaubühne am Lehniner Platz |
| 27 | Theatre | Wassa Schelesnowa [Vassa Zheleznova] | Deutsches Theater Berlin |
| 9 | Poetry slam | Insel Slam – Poetry | Insel am Treptower Park |
| 8 | Reading | Blanche Kommerell reads Franz Kafka | Literaturhaus Berlin |
| 20 | Reading | Die Kriegstagebücher Erich Mühsams  [Erich Mühsam’s war diaries] | Museum Neukölln |
| 28 | Exhibition | 8th Berlin Biennale for Contemporary Art | Museen Dahlem of the National Museums in Berlin |
| 30 | Exhibition | Ai Weiwei: Evidence | Martin-Gropius-Bau of the National Museums in Berlin |
| 20 | Exhibition | Collection of old master paintings | Gemäldegalerie (Old Master Paintings) of the National Museums in Berlin |
| 22 | Exhibition | Egyptian museum, papyrus collection, and museum of prehistory and early history | New Museum of the National Museums in Berlin |
| 20 | Film | Stereo | Open air cinema at Kulturforum am Potsdamer Platz |
| 10 | Film | Finding Vivian Maier | Filmtheater am Friedrichshain |
| 15 | Film | Boyhood | Filmtheater am Friedrichshain |
| 9 | Film | The Grand Budapest Hotel | B-Ware Ladenkino |
| 28 | Film | Monsieur Claude und seine Töchter [Monsieur Claude and his daughters] | Filmtheater am Friedrichshain |

*Note*. *n* indicates the number of completed questionnaires per event.
