# Supplementary material for: Measuring aesthetic emotions: A review of the literature and a new assessment tool
Source: PLoS One. 2017 Jun 5;12(6):e0178899. doi: 10.1371/journal.pone.0178899 (PMC5459466; doi:10.1371/journal.pone.0178899)
Supplement: S3 Table — (DOCX) [file pone.0178899.s005.docx]

**S3 Table. Factor Structure Matrix of an Exploratory Factor Analysis with 24 Factors.**

|  | **Factor** | | | | | | | | | | | | | | | | | | | | | | | |
| --- | --- | --- | --- | --- | --- | --- | --- | --- | --- | --- | --- | --- | --- | --- | --- | --- | --- | --- | --- | --- | --- | --- | --- | --- |
| **Item (subscale number)** | **1** | **2** | **3** | **4** | **5** | **6** | **7** | **8** | **9** | **10** | **11** | **12** | **13** | **14** | **15** | **16** | **17** | **18** | **19** | **20** | **21** | **22** | **23** | **24** |
| **3 Invigorated me (9)** | **.88** | -.25 | .05 | .24 | -.26 | **.36** | .02 | **.34** | **.42** | **.30** | .22 | .15 | .18 | -.16 | **.50** | **.38** | .25 | **.31** | -.18 | .29 | -.23 | -.21 | -.10 | .23 |
| **2 Spurred me on (9)** | **.81** | **-.32** | .06 | .15 | -.27 | **.34** | .08 | **.32** | **.39** | .17 | .29 | .23 | .23 | -.14 | **.48** | .26 | .29 | **.30** | -.22 | **.50** | -.18 | **-.31** | -.06 | .08 |
| 1 Filled me with longing | **.46** | -.10 | .08 | .10 | -.13 | .27 | .13 | .14 | .26 | .08 | .26 | .21 | **.53** | -.14 | **.35** | .18 | .25 | .27 | -.12 | **.50** | .10 | **-.31** | .24 | -.13 |
| 22 Felt depressed | **-.38** | **.83** | .26 | .21 | **.51** | -.08 | **.45** | -.01 | -.24 | .17 | -.10 | .23 | .03 | **.50** | -.09 | **-.30** | **-.35** | .06 | **.45** | -.12 | -.05 | .29 | **.45** | .06 |
| **5 Felt oppressive (20)** | -.28 | **.70** | .28 | .17 | **.44** | -.13 | **.31** | -.11 | -.15 | .09 | -.06 | .11 | -.17 | **.49** | -.20 | **-.34** | **-.39** | .10 | **.40** | -.21 | .00 | .27 | **.41** | .11 |
| 21 Made me feel uncomfortable | **-.39** | **.61** | .12 | -.18 | **.61** | -.15 | **.38** | -.14 | **-.40** | .09 | -.16 | .07 | .07 | **.46** | -.12 | -.16 | -.23 | -.14 | **.52** | -.18 | .26 | **.40** | .16 | -.07 |
| **16 Worried me (20)** | -.29 | **.64** | **.34** | .29 | **.47** | -.06 | **.37** | -.16 | -.19 | .23 | -.11 | .19 | -.01 | **.51** | -.02 | -.28 | -.25 | .13 | **.45** | -.10 | -.06 | .18 | **.51** | .03 |
| **17 Challenged me intellectually (14)** | .03 | .21 | **.84** | .09 | .18 | .02 | **.31** | .05 | .08 | .26 | .11 | **.30** | .08 | .18 | .17 | -.12 | -.07 | **.35** | .18 | .09 | -.05 | .03 | .21 | .11 |
| **24 Was mentally engaged (14)** | .09 | .13 | **.83** | .15 | .02 | .16 | .24 | .23 | .20 | .29 | .15 | **.38** | .23 | .14 | .12 | -.06 | -.03 | **.34** | .13 | .11 | -.11 | .03 | .22 | .21 |
| **23 Felt a sudden insight (15)** | .10 | .18 | **.52** | .16 | .10 | .16 | **.36** | .11 | .16 | .28 | .04 | **.50** | .27 | .19 | **.32** | .04 | .05 | **.38** | .00 | **.31** | -.09 | .09 | **.32** | **.30** |
| **15 Felt deeply moved (3)** | .25 | .12 | .20 | **.80** | -.02 | **.35** | .08 | **.41** | **.47** | **.36** | .19 | **.34** | .22 | .01 | **.30** | .02 | -.02 | **.33** | .00 | **.33** | **-.33** | -.12 | **.33** | .27 |
| 49 Was overwhelmed | **.30** | -.02 | .17 | **.60** | .02 | .25 | .18 | **.44** | **.43** | **.54** | **.41** | **.36** | .29 | .08 | **.40** | .10 | .17 | **.38** | .07 | **.58** | -.21 | -.13 | .15 | **.33** |
| 6 Gripped me | **.39** | .04 | .25 | **.59** | -.10 | **.37** | .10 | **.39** | **.53** | **.42** | .15 | .20 | .08 | .07 | **.33** | .14 | .13 | **.37** | .00 | .25 | **-.42** | -.09 | .17 | **.43** |
| 62 Felt that time was flying | **.38** | -.04 | .03 | **.48** | -.20 | **.31** | .12 | **.35** | **.35** | .28 | .01 | .04 | .12 | .06 | **.37** | .26 | .15 | **.35** | -.16 | .17 | **-.41** | -.18 | .08 | **.40** |
| **8 Made me angry (19)** | **-.33** | **.38** | .07 | .00 | **.84** | -.22 | .24 | -.20 | **-.33** | .08 | -.15 | .05 | -.09 | **.39** | -.21 | -.22 | **-.38** | -.09 | **.35** | -.19 | .23 | **.36** | .28 | -.11 |
| **35 Made me aggressive (19)** | -.24 | **.43** | .15 | .06 | **.81** | -.12 | **.32** | -.19 | **-.30** | .03 | -.20 | .16 | -.08 | **.46** | -.08 | -.25 | **-.36** | .03 | **.47** | -.12 | .14 | **.41** | .24 | .02 |
| 36 Disliked it | **-.43** | **.38** | .03 | -.26 | **.70** | -.19 | .27 | **-.32** | **-.57** | -.03 | -.09 | -.04 | .01 | **.46** | -.24 | -.23 | **-.32** | **-.34** | **.48** | -.27 | **.50** | **.49** | -.02 | -.10 |
| 56 Scared me | -.29 | **.48** | .17 | **.30** | **.64** | -.15 | **.30** | -.07 | -.20 | .27 | -.14 | .12 | .02 | **.50** | -.06 | -.22 | **-.36** | .10 | **.61** | -.17 | -.09 | **.34** | **.45** | .10 |
| **12 Touched me (3)** | **.35** | .09 | .27 | **.58** | -.04 | **.58** | .04 | **.40** | **.54** | **.33** | .15 | **.35** | .25 | -.05 | **.30** | .09 | .08 | **.36** | -.06 | **.30** | **-.38** | -.16 | **.36** | **.42** |
| **13 Delighted me (7)** | **.62** | **-.41** | -.04 | .16 | **-.46** | **.59** | -.07 | **.39** | **.45** | .15 | .15 | .07 | .23 | -.29 | **.35** | **.54** | **.56** | .20 | **-.34** | **.32** | -.19 | -.25 | **-.30** | .08 |
| 18 Was not aware of myself | .04 | .20 | .24 | .01 | .20 | .04 | **.82** | .06 | -.03 | .18 | .19 | **.31** | .23 | .25 | .16 | .00 | .07 | .08 | .24 | .13 | .16 | .15 | .17 | .05 |
| **19 Was impressed (2)** | **.39** | .00 | .28 | **.42** | -.18 | .29 | .21 | **.66** | **.59** | **.41** | **.33** | **.35** | .16 | -.07 | .28 | .05 | .17 | **.46** | -.02 | **.32** | **-.32** | -.10 | .19 | **.33** |
| 14 I found it perfect | **.43** | .05 | .18 | **.48** | -.20 | **.40** | .12 | **.66** | **.44** | **.37** | .24 | **.41** | .29 | .02 | **.39** | .14 | .28 | **.38** | -.15 | **.38** | -.18 | -.17 | .11 | .15 |
| **28 I found it beautiful (1)** | **.45** | **-.37** | -.03 | .23 | **-.43** | **.41** | -.03 | **.40** | **.75** | .19 | **.38** | .18 | **.32** | -.20 | **.33** | **.34** | **.52** | **.33** | -.27 | **.35** | -.15 | **-.42** | -.09 | .16 |
| 29 Was attracted | **.47** | -.19 | .22 | **.38** | -.29 | **.35** | .03 | **.36** | **.72** | **.36** | .20 | **.33** | .26 | -.13 | **.45** | .18 | .28 | **.45** | -.21 | **.37** | -.28 | **-.31** | .05 | **.31** |
| **25 Liked it (1)** | **.44** | -.18 | .15 | **.44** | **-.37** | **.40** | -.07 | **.51** | **.83** | **.38** | .23 | .05 | .16 | -.28 | **.38** | **.34** | .28 | **.47** | -.26 | **.32** | **-.45** | -.26 | .13 | .27 |
| 30 Made me feel enthusiastic | **.53** | -.17 | .17 | **.42** | -.29 | **.36** | .01 | **.54** | **.78** | **.46** | .13 | .21 | .24 | -.08 | **.46** | **.37** | **.33** | **.37** | -.22 | **.51** | **-.37** | -.19 | .03 | **.33** |
| **44 Surprised me (12)** | .25 | .04 | .21 | .26 | .02 | .29 | .20 | .29 | **.30** | **.80** | .14 | .16 | .12 | .14 | .29 | **.32** | .07 | **.38** | .17 | .17 | -.16 | -.04 | .06 | .26 |
| **31 Baffled me (12)** | .27 | .05 | .26 | **.38** | .05 | .15 | .24 | **.40** | **.37** | **.79** | .16 | .29 | .21 | .10 | .22 | .15 | .11 | **.36** | .17 | **.33** | -.18 | .00 | .14 | .22 |
| 57 Astonished me | .28 | -.20 | **.34** | .26 | .02 | .18 | .21 | **.57** | **.37** | **.65** | **.35** | **.30** | .16 | .13 | .27 | .13 | .11 | **.43** | .11 | .29 | -.25 | -.09 | .17 | .20 |
| **9 I found it sublime (4)** | .29 | -.11 | .26 | .12 | -.14 | .14 | .28 | .26 | .23 | .11 | **.75** | **.36** | .25 | -.01 | .19 | .02 | .23 | .10 | -.06 | .28 | .09 | .07 | -.04 | .01 |
| 48 I found it graceful | **.31** | -.12 | .01 | .16 | -.20 | .24 | .15 | .23 | .25 | .23 | **.66** | **.36** | **.42** | -.01 | **.31** | .16 | .25 | .27 | -.06 | **.38** | .07 | -.23 | .00 | .22 |
| 72 I found it harmonious | **.44** | -.23 | -.02 | .13 | **-.35** | .22 | .05 | **.32** | **.41** | .21 | **.48** | .17 | .25 | -.24 | **.39** | **.35** | **.51** | .29 | -.23 | .26 | -.11 | **-.46** | -.13 | **.35** |
| 32 Felt humbled | .15 | .14 | **.36** | .20 | .11 | .11 | **.33** | .21 | .14 | .19 | .26 | **.86** | **.30** | .07 | .23 | -.16 | .11 | **.32** | .05 | .22 | -.10 | -.03 | .26 | .17 |
| **51 Felt awe (4)** | .22 | -.02 | .16 | .28 | .04 | **.33** | **.39** | .25 | .24 | **.35** | **.47** | **.71** | **.34** | .11 | **.31** | -.12 | .06 | **.31** | .14 | .28 | .02 | -.16 | .21 | .26 |
| **45 Sensed a deeper meaning (15)** | .22 | .14 | **.45** | **.42** | .01 | **.38** | .25 | .17 | **.33** | **.47** | .10 | **.54** | .27 | .21 | **.33** | .03 | .09 | **.47** | -.11 | .24 | -.18 | -.11 | **.32** | **.36** |
| **41 Made me feel sentimental (6)** | .19 | -.01 | .11 | .15 | -.06 | .20 | .21 | .14 | .19 | .12 | .21 | .28 | **.88** | .01 | .27 | .20 | .23 | .15 | .05 | **.34** | .02 | -.07 | .26 | .15 |
| **33 Made me feel nostalgic (6)** | .17 | -.04 | .20 | .05 | -.05 | .11 | .26 | .11 | .12 | .16 | .26 | **.36** | **.76** | .05 | .23 | .19 | .28 | .26 | -.03 | **.30** | .16 | -.20 | .16 | -.03 |
| 27 Repelled me | **-.32** | **.46** | .10 | -.08 | **.56** | -.13 | **.39** | -.12 | **-.34** | .05 | -.04 | .00 | .01 | **.81** | -.13 | -.06 | -.21 | -.18 | **.51** | -.13 | .24 | **.50** | .13 | -.06 |
| 26 Was shocking to me | -.22 | **.45** | .27 | **.34** | **.49** | -.04 | **.35** | .07 | .02 | **.39** | -.07 | .13 | -.03 | **.64** | -.04 | -.10 | **-.35** | .20 | **.47** | -.12 | -.08 | **.39** | **.40** | .08 |
| **39 Energized me (10)** | **.46** | -.08 | .15 | .21 | -.15 | .24 | .18 | .27 | **.33** | .15 | .20 | .25 | .26 | -.03 | **.80** | .18 | .21 | .27 | -.07 | **.35** | -.21 | -.13 | .02 | .18 |
| 52 Perked me up | **.45** | -.22 | -.10 | .28 | -.26 | **.42** | .06 | .23 | **.33** | **.34** | .18 | .05 | .19 | -.06 | **.65** | **.48** | **.30** | .29 | -.03 | **.30** | -.26 | -.25 | -.10 | **.30** |
| **53 Motivated me to act (10)** | **.47** | -.15 | .09 | .13 | -.05 | **.32** | .18 | .12 | .23 | .27 | .13 | **.30** | **.33** | .01 | **.68** | .20 | **.30** | **.45** | -.09 | **.46** | -.02 | -.23 | .08 | .23 |
| 54 Felt absorbed in the experience | **.40** | -.16 | .26 | .20 | -.08 | .26 | **.37** | .16 | .26 | .23 | .14 | **.30** | **.47** | .18 | **.62** | .20 | .20 | **.43** | -.05 | **.44** | -.11 | -.15 | .16 | .21 |
| 42 Inspired me | **.54** | -.12 | **.36** | .11 | -.16 | **.31** | .10 | **.30** | **.42** | **.31** | .22 | **.43** | **.40** | .00 | **.63** | .21 | .23 | **.58** | -.13 | **.45** | -.08 | -.20 | .16 | **.31** |
| **59 Was funny to me (8)** | .26 | -.14 | -.15 | .03 | -.12 | .15 | .04 | .09 | .14 | .18 | -.03 | -.18 | .18 | .04 | .12 | **.91** | .17 | .10 | -.03 | .08 | -.02 | .05 | -.12 | .13 |
| **71 Amused me (8)** | **.31** | -.19 | -.05 | -.02 | -.21 | .20 | -.01 | .16 | .25 | .19 | .07 | -.16 | .23 | -.05 | .29 | **.91** | .28 | .11 | -.04 | .19 | -.11 | -.11 | -.14 | .04 |
| 43 Made me merry | **.52** | -.29 | -.07 | .06 | **-.30** | **.45** | -.06 | .19 | **.40** | .27 | .15 | .01 | .27 | -.21 | **.44** | **.74** | **.43** | .27 | -.16 | **.32** | -.15 | -.21 | -.22 | .11 |
| 4 Made me cheerful | **.63** | **-.39** | -.15 | .08 | **-.43** | **.46** | -.13 | .27 | **.45** | .14 | .16 | -.06 | .27 | -.29 | **.35** | **.65** | **.53** | .18 | -.25 | **.32** | -.10 | -.23 | **-.33** | .04 |
| **7 Calmed me (11)** | .15 | -.20 | .02 | -.07 | -.24 | .11 | .11 | .14 | .22 | .03 | .25 | .22 | **.31** | -.17 | .17 | .07 | **.68** | .11 | -.18 | .23 | .06 | -.11 | -.08 | -.03 |
| **74 Relaxed me (11)** | **.35** | **-.31** | -.19 | .10 | **-.43** | .24 | .00 | .24 | **.36** | .13 | .18 | .14 | .28 | -.20 | **.32** | **.40** | **.71** | .13 | **-.30** | .23 | .06 | **-.30** | -.21 | .27 |
| 10 Made me feel content | **.53** | **-.30** | .01 | .10 | **-.46** | **.39** | -.06 | **.36** | **.48** | .05 | .28 | .07 | .24 | -.14 | **.37** | **.34** | **.67** | .20 | -.25 | **.37** | -.23 | -.21 | **-.30** | .05 |
| **11 Made me happy (7)** | **.62** | **-.32** | -.02 | .18 | **-.45** | **.53** | -.03 | **.38** | **.52** | .14 | **.31** | .05 | .28 | -.17 | **.41** | **.48** | **.66** | .23 | -.25 | **.49** | -.21 | -.27 | **-.35** | .09 |
| 55 I found it pleasant | **.48** | **-.49** | -.05 | .01 | **-.50** | **.32** | -.03 | **.40** | **.55** | .09 | **.30** | .00 | .27 | -.27 | **.41** | **.43** | **.65** | **.38** | **-.34** | .25 | -.17 | **-.40** | -.13 | .08 |
| **34 Made me curious (13)** | .23 | .03 | **.41** | .19 | -.01 | .13 | .06 | .24 | .29 | **.36** | .08 | **.36** | .18 | -.03 | .26 | .07 | .08 | **.77** | .00 | .21 | -.15 | -.15 | .17 | .27 |
| **46 Sparked my interest (13)** | .29 | -.02 | **.34** | **.41** | -.11 | **.42** | .16 | **.39** | **.58** | **.48** | .21 | **.33** | .10 | .08 | **.42** | .14 | .13 | **.73** | -.10 | .25 | **-.35** | -.17 | .19 | **.39** |
| **61 Felt confused (18)** | -.19 | **.35** | .22 | .07 | **.47** | -.09 | **.41** | -.06 | -.19 | .27 | -.01 | .11 | .08 | **.44** | -.04 | -.04 | -.23 | .03 | **.84** | -.05 | .16 | **.35** | .20 | .05 |
| **69 Was unsettling to me (18)** | **-.32** | **.53** | .22 | .10 | **.41** | .11 | **.37** | -.11 | -.24 | .22 | -.10 | .10 | -.04 | **.42** | -.11 | -.24 | **-.36** | -.01 | **.77** | -.08 | .12 | **.39** | **.43** | -.07 |
| 40 Irritated me | -.22 | **.36** | .24 | -.13 | **.48** | -.09 | **.34** | -.13 | **-.30** | .19 | .02 | .14 | .06 | **.40** | -.06 | -.07 | -.20 | .00 | **.70** | -.13 | .22 | **.44** | .12 | -.01 |
| 60 I found it unpleasant | **-.40** | **.41** | .01 | -.15 | **.57** | -.28 | .24 | -.22 | **-.42** | .03 | -.11 | -.02 | .00 | **.47** | -.16 | -.16 | -.26 | -.17 | **.70** | -.19 | .23 | **.58** | .13 | -.10 |
| **50 Was enchanted (5)** | **.51** | -.21 | .08 | **.30** | -.25 | **.36** | .20 | **.41** | **.48** | **.38** | **.42** | **.33** | **.45** | -.08 | **.46** | **.31** | **.36** | **.34** | -.11 | **.75** | -.14 | -.23 | .00 | .21 |
| **37 Felt something wonderful (5)** | **.54** | -.23 | .11 | .24 | -.28 | **.42** | .17 | **.42** | **.49** | .18 | **.31** | .27 | **.41** | -.11 | **.54** | **.30** | **.36** | **.37** | -.22 | **.68** | -.10 | -.22 | -.11 | .14 |
| 70 Put me in a dreamy mood | **.35** | -.24 | .01 | .01 | **-.32** | .22 | .14 | .11 | .24 | .08 | **.30** | .21 | **.59** | -.22 | **.38** | **.34** | **.44** | .17 | -.07 | **.58** | .10 | -.25 | .03 | .18 |
| 65 Was enraptured | **.41** | -.14 | .08 | **.49** | -.20 | **.36** | .15 | **.51** | **.44** | **.43** | .29 | .24 | .28 | .06 | **.50** | .26 | .16 | **.34** | -.08 | **.59** | -.29 | -.17 | .15 | **.35** |
| **64 Felt indifferent (17)** | **-.35** | .13 | -.13 | **-.31** | **.33** | **-.31** | .28 | -.24 | **-.47** | -.21 | -.03 | -.15 | -.02 | **.33** | -.29 | -.14 | -.14 | **-.30** | **.31** | -.18 | **.74** | **.32** | -.13 | **-.33** |
| **68 Bored me (17)** | **-.38** | .08 | -.13 | **-.45** | **.37** | **-.31** | .11 | **-.42** | **-.45** | -.25 | .07 | -.18 | -.05 | .07 | **-.39** | -.19 | -.15 | **-.35** | .27 | -.18 | **.79** | **.49** | -.09 | -.24 |
| 38 Tired me | **-.31** | .08 | .07 | **-.36** | **.36** | -.22 | .19 | **-.34** | **-.47** | -.23 | .14 | .03 | .10 | .19 | -.26 | -.24 | .00 | -.24 | .28 | -.21 | **.71** | **.30** | -.05 | -.20 |
| **58 I found it ugly (16)** | **-.36** | **.32** | .08 | -.10 | **.56** | -.20 | .29 | -.15 | **-.35** | .04 | -.02 | -.02 | -.12 | **.43** | -.20 | -.14 | -.22 | -.26 | **.47** | -.21 | **.30** | **.82** | .06 | -.11 |
| **63 I found it distasteful (16)** | -.26 | .29 | -.16 | -.13 | **.41** | -.14 | **.32** | -.19 | **-.38** | -.04 | -.07 | -.13 | -.04 | **.50** | -.22 | .04 | -.19 | -.15 | **.50** | -.18 | **.41** | **.72** | -.03 | -.17 |
| **67 Made me sad (21)** | -.12 | **.39** | .26 | .28 | **.35** | .06 | **.36** | .04 | .01 | .14 | -.04 | **.31** | **.31** | **.32** | .05 | -.16 | -.26 | .19 | .29 | .04 | -.09 | .11 | **.83** | .21 |
| **20 Made me feel melancholic (21)** | -.06 | **.34** | .15 | **.32** | .19 | .04 | .28 | .07 | .08 | .18 | .03 | **.36** | **.41** | .05 | .09 | -.07 | -.02 | .22 | .17 | .18 | -.10 | -.02 | **.67** | .18 |
| 73 Stimulated my thoughts | .29 | .01 | **.39** | .28 | -.15 | .26 | .12 | .20 | **.37** | .30 | .14 | **.34** | .23 | -.04 | **.35** | .10 | .10 | **.53** | -.10 | .26 | -.23 | -.21 | **.30** | **.67** |
| **75 Fascinated me (2)** | **.39** | -.10 | .25 | **.39** | -.16 | **.35** | .17 | **.60** | **.57** | **.52** | .23 | **.37** | .22 | .05 | **.47** | .23 | .22 | **.52** | -.07 | **.47** | -.24 | -.25 | .12 | **.55** |
| 66 Moved me | **.31** | .07 | .23 | **.45** | -.09 | **.49** | .16 | **.43** | **.47** | **.37** | .16 | .30 | .24 | .07 | **.46** | .14 | .00 | **.42** | -.02 | **.43** | **-.45** | -.24 | **.40** | **.54** |
| 47 Agitated me | .08 | **.40** | .25 | **.43** | .25 | .26 | **.41** | .10 | .15 | .25 | .09 | **.34** | .23 | **.32** | .20 | -.09 | -.17 | **.34** | **.36** | .25 | -.24 | .15 | **.36** | **.41** |

*Note.* The 42 items included in the final AESTHEMOS along with the respective subscale number and loadings with λ ≥ |.30| are highlighted in bold.
