# Supplementary material for: Measuring aesthetic emotions: A review of the literature and a new assessment tool
Source: PLoS One. 2017 Jun 5;12(6):e0178899. doi: 10.1371/journal.pone.0178899 (PMC5459466; doi:10.1371/journal.pone.0178899)
Supplement: S4 Table — (DOCX) [file pone.0178899.s006.docx]

**S4 Table. Factor Correlation Matrix Based on an EFA with 24 Factors and Oblimin Rotation.**

| **Factor** | **1** | **2** | **3** | **4** | **5** | **6** | **7** | **8** | **9** | **10** | **11** | **12** | **13** | **14** | **15** | **16** | **17** | **18** | **19** | **20** | **21** | **22** | **23** |
| --- | --- | --- | --- | --- | --- | --- | --- | --- | --- | --- | --- | --- | --- | --- | --- | --- | --- | --- | --- | --- | --- | --- | --- |
| 1 Vitality/arousal |  |  |  |  |  |  |  |  |  |  |  |  |  |  |  |  |  |  |  |  |  |  |  |
| 2 Uneasiness/fear | **-.27** |  |  |  |  |  |  |  |  |  |  |  |  |  |  |  |  |  |  |  |  |  |  |
| 3 Intellectual challenge | .04 | .16 |  |  |  |  |  |  |  |  |  |  |  |  |  |  |  |  |  |  |  |  |  |
| 4 Being moved | .17 | .15 | .07 |  |  |  |  |  |  |  |  |  |  |  |  |  |  |  |  |  |  |  |  |
| 5 Anger | **-.27** | **.36** | .10 | -.01 |  |  |  |  |  |  |  |  |  |  |  |  |  |  |  |  |  |  |  |
| 6 Joyfully moved | **.34** | -.04 | .06 | .24 | -.18 |  |  |  |  |  |  |  |  |  |  |  |  |  |  |  |  |  |  |
| 7 Flow/absorption | .00 | **.23** | **.21** | .07 | **.23** | .04 |  |  |  |  |  |  |  |  |  |  |  |  |  |  |  |  |  |
| 8 Captivation | **.28** | -.10 | .10 | .29 | -.17 | .20 | .06 |  |  |  |  |  |  |  |  |  |  |  |  |  |  |  |  |
| 9 Feeling of beauty | **.38** | -.18 | **.12** | .34 | **-.31** | .33 | -.05 | **.42** |  |  |  |  |  |  |  |  |  |  |  |  |  |  |  |
| 10 Surprise | **.15** | .04 | **.22** | **.31** | .09 | .17 | **.18** | **.31** | **.27** |  |  |  |  |  |  |  |  |  |  |  |  |  |  |
| 11 Sublimity/harmony | **.20** | -.15 | .07 | .08 | **-.12** | .12 | **.17** | .22 | .21 | .13 |  |  |  |  |  |  |  |  |  |  |  |  |  |
| 12 Awe | .14 | .10 | **.31** | .19 | .08 | .15 | **.29** | .17 | .12 | **.22** | **.24** |  |  |  |  |  |  |  |  |  |  |  |  |
| 13 Nostalgia/longing | **.19** | -.03 | **.10** | .08 | -.04 | **.17** | **.22** | .10 | .13 | .09 | **.22** | **.31** |  |  |  |  |  |  |  |  |  |  |  |
| 14 Repulsion | -.17 | **.32** | **.13** | .09 | **.39** | -.02 | **.35** | -.01 | -.17 | .15 | -.05 | .07 | .01 |  |  |  |  |  |  |  |  |  |  |
| 15 Energy | **.44** | -.12 | **.11** | **.17** | **-.13** | **.28** | .15 | .19 | **.27** | **.22** | **.14** | **.22** | **.26** | .01 |  |  |  |  |  |  |  |  |  |
| 16 Humor | **.33** | **-.20** | -.13 | .00 | **-.21** | .21 | -.01 | .14 | **.22** | **.18** | .02 | **-.17** | **.21** | -.05 | **.23** |  |  |  |  |  |  |  |  |
| 17 Relaxation | **.28** | **-.24** | -.07 | -.04 | **-.32** | .18 | .02 | **.17** | **.28** | .03 | **.21** | .08 | **.27** | **-.18** | **.23** | **.29** |  |  |  |  |  |  |  |
| 18 Interest | **.27** | -.02 | **.31** | **.23** | -.06 | **.20** | .12 | **.22** | **.35** | **.33** | .10 | **.29** | **.16** | .04 | **.33** | .11 | .11 |  |  |  |  |  |  |
| 19 Confusion | **-.22** | **.33** | **.09** | .00 | **.41** | -.05 | **.30** | -.08 | **-.22** | **.19** | .01 | .01 | .03 | **.35** | -.07 | -.07 | **-.21** | -.04 |  |  |  |  |  |
| 20 Enchantment/wonder | **.35** | -.12 | .07 | .19 | **-.15** | **.23** | .12 | .24 | **.29** | **.18** | **.23** | **.23** | **.35** | -.06 | **.37** | **.15** | **.20** | **.22** | -.08 |  |  |  |  |
| 21 Boredom | **-.17** | -.01 | -.10 | **-.33** | **.19** | **-.16** | .14 | -.25 | **-.31** | **-.14** | .10 | -.02 | .12 | .09 | **-.18** | -.07 | .02 | **-.18** | .13 | -.06 |  |  |  |
| 22 Feeling of ugliness | **-.22** | **.24** | .05 | -.07 | **.36** | -.12 | **.18** | -.10 | **-.22** | -.02 | -.08 | -.06 | -.11 | **.30** | **-.19** | -.06 | **-.19** | **-.18** | **.34** | **-.14** | .20 |  |  |
| 23 Sadness | -.12 | **.30** | **.21** | **.25** | **.21** | .02 | **.21** | .01 | .05 | .13 | -.05 | **.22** | **.21** | **.13** | .03 | **-.18** | **-.23** | **.20** | **.16** | .05 | **-.13** | .00 |  |
| 24 Preoccupation | .11 | .03 | **.12** | **.25** | -.04 | .17 | .07 | .14 | .22 | **.24** | .06 | **.20** | .08 | .03 | **.22** | .09 | .01 | **.26** | -.02 | .11 | **-.19** | -.08 | .14 |

*Note*. Correlations with *p < .05* are printed in bold.
