# Supplementary material for: Measuring aesthetic emotions: A review of the literature and a new assessment tool
Source: PLoS One. 2017 Jun 5;12(6):e0178899. doi: 10.1371/journal.pone.0178899 (PMC5459466; doi:10.1371/journal.pone.0178899)
Supplement: S5 Table — (DOCX) [file pone.0178899.s007.docx]

**S5 Table. Factor Structure Matrix of an Exploratory Factor Analysis with Seven Factors.**

| **Factor** | **1** | **2** | **3** | **4** | **5** | **6** | **7** | **Subscale (number)** |
| --- | --- | --- | --- | --- | --- | --- | --- | --- |
| *Factor 1: Negative emotions* |  |  |  |  |  |  |  |  |
| **63 I found it distasteful** | **.76** | -.22 | -.15 | -.29 | .01 | -.05 | -.07 | Feeling of ugliness (16) |
| **58 I found it ugly** | **.78** | -.19 | -.01 | **-.35** | -.09 | .02 | -.24 | Feeling of ugliness (16) |
| 27 Repelled me | **.81** | -.17 | .08 | -.22 | -.05 | .19 | -.17 | -- |
| 60 I found it unpleasant | **.82** | -.26 | .00 | **-.34** | -.07 | .16 | -.23 | -- |
| 36 Disliked it | **.81** | **-.43** | -.07 | **-.37** | .02 | .03 | **-.35** | -- |
| 21 Made me feel uncomfortable | **.77** | -.24 | .12 | -.27 | -.01 | .26 | -.25 | -- |
| **61 Felt confused** | **.75** | .04 | .28 | -.13 | -.04 | .27 | -.07 | Confusion (18) |
| **69 Was unsettling to me** | **.73** | -.01 | .26 | -.24 | -.14 | **.40** | -.24 | Confusion (18) |
| 26 Was shocking to me | **.62** | .20 | **.39** | -.12 | **-.30** | **.49** | -.14 | -- |
| 40 Irritated me | **.74** | -.10 | .25 | -.17 | .01 | .14 | -.13 | -- |
| **35 Made me aggressive** | **.72** | -.18 | .16 | -.13 | -.23 | **.33** | **-.34** | Anger (19) |
| **8 Made me angry** | **.69** | -.24 | .06 | -.26 | -.19 | .28 | **-.36** | Anger (19) |
| **64 Felt indifferent** | **.59** | **-.44** | -.24 | **-.37** | .26 | -.23 | -.27 | Boredom (17) |
| **68 Bored me** | **.54** | **-.50** | -.28 | **-.46** | .27 | -.29 | **-.32** | Boredom (17) |
| 38 Tired me | **.51** | **-.44** | -.08 | **-.32** | **.40** | -.21 | **-.35** | -- |
| **16 Worried me** | **.58** | .03 | **.39** | -.15 | -.19 | **.58** | **-.31** | Uneasiness (20) |
| **5 Felt oppressive** | **.57** | -.01 | **.32** | -.28 | -.29 | **.46** | **-.40** | Uneasiness (20) |
| 56 Scared me | **.69** | .03 | .26 | -.17 | -.29 | **.56** | -.24 | -- |
| 22 Felt depressed | **.66** | .01 | **.35** | -.22 | -.17 | **.60** | **-.37** | -- |
| 18 Was not aware of myself | **.37** | .13 | **.33** | .19 | .28 | .18 | -.05 | -- |
| 55 I found it pleasant | **-.61** | **.44** | .13 | **.50** | **.38** | -.21 | **.57** | -- |
| *Factor 2: Prototypical aesthetic emotions* | | | | | | | | |
| **15 Felt deeply moved** | -.15 | **.69** | **.34** | **.41** | -.07 | **.53** | .07 | Being moved (3) |
| **12 Touched me** | -.25 | **.66** | **.43** | **.46** | -.05 | **.52** | .17 | Being moved (3) |
| **31 Baffled me** | .10 | **.66** | **.50** | **.34** | .02 | .21 | .26 | Surprise (12) |
| **44 Surprised me** | .11 | **.56** | **.47** | **.34** | -.07 | .13 | **.41** | Surprise (12) |
| 57 Astonished me | -.02 | **.66** | **.53** | **.39** | .06 | .14 | .21 | -- |
| **19 Was impressed** | -.23 | **.76** | **.50** | **.44** | .05 | .23 | .15 | Fascination (2) |
| **75 Fascinated me** | -.25 | **.75** | **.54** | **.61** | .07 | .27 | **.33** | Fascination (2) |
| 49 Was overwhelmed | -.06 | **.78** | **.37** | **.55** | .15 | **.32** | .17 |  |
| 6 Gripped me | -.19 | **.67** | **.41** | **.45** | -.17 | **.33** | .25 | -- |
| 30 Made me feel enthusiastic | **-.41** | **.76** | **.36** | **.62** | .06 | .15 | **.50** | -- |
| **25 Liked it** | **-.54** | **.73** | **.34** | **.47** | -.02 | .18 | **.50** | Feeling of beauty/liking (1) |
| **28 I found it beautiful** | **-.57** | **.61** | .18 | **.49** | **.36** | -.06 | **.48** | Feeling of beauty/liking (1) |
| 29 Was attracted | **-.44** | **.65** | **.43** | **.57** | .13 | .16 | **.33** | -- |
| 65 Was enraptured | -.22 | **.72** | .27 | **.65** | .10 | .28 | **.32** | -- |
| 72 I found it harmonious | **-.45** | **.47** | .19 | **.50** | **.35** | -.08 | **.41** | -- |
| 14 I found it perfect | -.24 | **.68** | **.37** | **.54** | .17 | .22 | .21 | -- |
| **9 I found it sublime** | -.05 | **.42** | .25 | **.33** | **.46** | -.11 | -.04 | Awe (4) |
| **51 Felt awe** | .04 | **.51** | **.44** | **.45** | **.37** | .27 | -.14 | Awe (4) |
| 10 Made me feel content | **-.47** | **.50** | .05 | **.51** | **.34** | -.28 | **.51** | -- |

| **Factor** | **1** | **2** | **3** | **4** | **5** | **6** | **7** | **Subscale (number)** |
| --- | --- | --- | --- | --- | --- | --- | --- | --- |
| *Factor 3: Epistemic emotions* |  |  |  |  |  |  |  |  |
| **17 Challenged me intellectually** | .19 | .13 | **.78** | .13 | .03 | .20 | -.15 | Intellectual challenge (14) |
| **24 Was mentally engaged** | .08 | .27 | **.78** | .16 | .09 | .24 | -.06 | Intellectual challenge (14) |
| 73 Stimulated my thoughts | -.24 | **.43** | **.59** | **.46** | .03 | **.38** | .17 | -- |
| **34 Made me curious** | -.12 | **.36** | **.61** | **.37** | .04 | .23 | .13 | Interest (13) |
| **46 Sparked my interest** | -.23 | **.65** | **.63** | **.50** | -.07 | **.31** | .26 | Interest (13) |
| **23 Felt a sudden insight** | .11 | .25 | **.64** | **.36** | .16 | **.39** | .01 | Insight (15) |
| **45 Sensed a deeper meaning** | -.05 | **.48** | **.65** | **.42** | .07 | **.44** | .08 | Insight (15) |
| *Factor 4: Animation* |  |  |  |  |  |  |  |  |
| **53 Motivated me to act** | -.16 | **.32** | **.31** | **.74** | .26 | .15 | .28 | Energy (10) |
| **39 Energized me** | -.20 | **.36** | .27 | **.70** | .15 | .13 | .27 | Energy |
| **2 Spurred me on** | **-.39** | **.46** | .18 | **.77** | .22 | -.12 | **.32** | Vitality (9) |
| **3 Invigorated me** | **-.36** | **.50** | .20 | **.74** | .07 | -.09 | **.45** | Vitality |
| 52 Perked me up | -.27 | **.45** | .13 | **.64** | .05 | .05 | **.57** | -- |
| 42 Inspired me | -.24 | **.43** | **.57** | **.73** | .27 | .18 | .28 | -- |
| **37 Felt something wonderful** | **-.35** | **.57** | .22 | **.75** | **.38** | .03 | **.38** | Enchantment (5) |
| **50 Was enchanted** | -.26 | **.68** | .26 | **.72** | **.42** | .11 | **.36** | Enchantment (5) |
| 54 Felt absorbed in the experience | -.09 | **.32** | **.41** | **.68** | .30 | .25 | .27 | -- |
| 62 Felt that time was flying | -.26 | **.49** | .23 | **.46** | -.14 | .27 | **.36** | -- |
| *Factor 5: Nostalgia/relaxation* |  |  |  |  |  |  |  |  |
| **33 Made me feel nostalgic** | -.03 | .18 | .30 | **.34** | **.67** | .25 | .21 | Nostalgia (6) |
| **41 Made me feel sentimental** | -.03 | .23 | .22 | **.37** | **.59** | **.39** | .25 | Nostalgia (6) |
| 1 Filled me with longing | -.22 | .27 | .15 | **.53** | **.50** | .16 | .21 | -- |
| 70 Put me in a dreamy mood | -.27 | .27 | .10 | **.53** | **.62** | .05 | **.38** | -- |
| 48 I found it graceful | -.15 | **.49** | .20 | **.47** | **.49** | .03 | .15 | -- |
| **7 Calmed me** | -.26 | .21 | .09 | .24 | **.52** | -.17 | .19 | Relaxation (11) |
| **74 Relaxed me** | **-.45** | **.35** | .02 | **.40** | **.41** | -.19 | **.49** | Relaxation (11) |
| *Factor 6: Sadness* |  |  |  |  |  |  |  |  |
| **67 Made me sad** | **.34** | .11 | **.38** | .04 | .03 | **.80** | -.19 | Sadness (21) |
| **20 Made me feel melancholic** | .15 | .20 | **.31** | .11 | .18 | **.69** | -.08 | Sadness (21) |
| 66 Moved me | -.21 | **.63** | **.47** | **.56** | -.07 | **.55** | .22 | -- |
| 47 Agitated me | .29 | **.38** | **.44** | .29 | -.07 | **.59** | -.07 | -- |
| *Factor 7: Amusement* |  |  |  |  |  |  |  |  |
| **71 Amused me** | -.16 | .17 | -.01 | .30 | .14 | -.07 | **.88** | Humor (8) |
| **59 Was funny to me** | -.04 | .10 | -.05 | .18 | .06 | -.04 | **.82** | Humor (8) |
| 43 Made me merry | **-.35** | **.36** | .07 | **.55** | .21 | -.14 | **.79** | **--** |
| **13 Delighted me** | **-.53** | **.47** | .02 | **.57** | .25 | -.27 | **.67** | Joy (7) |
| **11 Made me happy** | **-.49** | **.59** | .04 | **.63** | **.36** | -.28 | **.63** | Joy (7) |
| 4 Made me cheerful | **-.48** | **.38** | -.07 | **.54** | .27 | -.29 | **.76** | -- |
| 32 Felt humbled | .06 | **.34** | **.54** | **.35** | **.32** | **.33** | -.23 | -- |

*Note.* The 42 items included in the final AESTHEMOS and loadings with λ ≥ |.30| are highlighted in bold.
