# Supplementary material for: Measuring aesthetic emotions: A review of the literature and a new assessment tool
Source: PLoS One. 2017 Jun 5;12(6):e0178899. doi: 10.1371/journal.pone.0178899 (PMC5459466; doi:10.1371/journal.pone.0178899)
Supplement: S6 Table — (DOCX) [file pone.0178899.s008.docx]

**S6 Table. Factor Correlation Matrix Based on an EFA with Seven Factors and Oblimin Rotation.**

| **Factor** | **1** | **2** | **3** | **4** | **5** | **6** |
| --- | --- | --- | --- | --- | --- | --- |
| 1 Negative emotions |  |  |  |  |  |  |
| 2 Prototypical aesthetic emotions | **-.23** |  |  |  |  |  |
| 3 Epistemic emotions | -.09 | **.37** |  |  |  |  |
| 4 Animation | **-.31** | **.50** | **.30** |  |  |  |
| 5 Nostalgia/relaxation | -.06 | .06 | .04 | **.26** |  |  |
| 6 Sadness | **.19** | **.20** | **.35** | .11 | **-.11** |  |
| 7 Amusement | **-.28** | **.28** | -.01 | **.36** | .10 | -.11 |

*Note*. Correlations with *p* < .05 are printed in bold.
